# Great Ape Childhoods: Social and Spatial Pathways to Independence in Bonobo and Chimpanzee Infants

**DOI:** 10.1111/desc.70102

**Published:** 2025-12-15

**Authors:** Jolinde M. R. Vlaeyen, Bas van Boekholt, Franziska Wegdell, Raymond Katumba, Andreas Berghänel, Martin Surbeck, Simone Pika

**Affiliations:** ^1^ Comparative BioCognition, Institute of Cognitive Science Osnabrück University Osnabrück Germany; ^2^ Department of Evolutionary Anthropology University of Zürich Zürich Switzerland; ^3^ Institute for the Interdisciplinary Study of Language Evolution University of Zürich Zürich Switzerland; ^4^ Sebitoli Chimpanzee Project, Great Ape Conservation Project Fort Portal Uganda; ^5^ Department of Interdisciplinary Life Sciences University of Veterinary Medicine Vienna Austria; ^6^ Department of Biology Zoology and Animal Ecology Research Group, University of Hildesheim Hildesheim Germany; ^7^ Department of Human Evolutionary Biology Harvard University Cambridge Massachusetts USA; ^8^ Department of Human Behaviour, Ecology and Culture Max Planck Institute for Evolutionary Anthropology Leipzig Germany

**Keywords:** developmental milestones, developmental trajectories, evolution, extended development, infant

## Abstract

Human development is marked by extended immaturity, necessitating extended care throughout infancy and childhood, facilitating advanced cognitive, social, and cultural skill acquisition. Parallels of extended development are also present in our closest living relatives, bonobos (*Pan paniscus*) and chimpanzees (*Pan troglodytes*), which differ markedly in their social systems. Bonobos live in more tolerant, female‐bonded societies, while societies of chimpanzees are more hierarchical, and male‐dominated. These differences in social ecology may thus also shape the pace and nature of early behavioural development. However, systematic, quantitative comparisons of developmental patterns of bonobos and chimpanzees are very limited, and results are conflicting. Hence, this study addressed this crucial gap by systematically examining behavioural development in bonobo and chimpanzee infants living in two populations (Kokolopori community, Kokolopori Bonobo Reserve, DRC, *N* = 21; Ngogo community, Kibale National Park, Uganda, *N* = 22) in their natural environments. We specifically focused on infants aged 0–5.5 years and investigated behavioural markers of independence (travel, feeding, grooming) and spatial independence. Our results showed similar developmental trajectories but marked species differences concerning specific social and spatial patterns. While chimpanzee infants exhibited prolonged dorsal riding, bonobo infants travelled independently more often and maintained greater distances from their mothers. In addition, age, sibling presence and maternal parity, but not sex, influenced behavioural patterns. These findings highlight the importance of systematic comparative developmental research across great ape populations for understanding both species‐specific adaptations and the broader evolutionary foundations of extended development in the human lineage.

## Summary

- Developmental trajectories were largely similar, but differences concerned spatial independence (chimpanzees stayed closer to mothers), possibly due to high‐risk exposure and maternal strategy differences.
- Differences were found concerning dorsal riding (longer in chimpanzees) and independent travel (more often in bonobos), possibly reflecting population‐specific maternal strategies and habitat structure differences.
- In both species, sibling births accelerated travel independence and reduced nipple contact, while maternal experience affected travelling patterns, highlighting possible protection and autonomy balance.
- Our findings underscore the importance of direct cross‐species comparisons of wild populations for understanding how social and spatial pathways contribute to behavioural diversity.

## 1 Introduction

Postnatal development is a critical phase in mammalian life history (Purvis and Harvey 1995), especially for altricial species such as primates. Unlike precocial species, essential traits (e.g., motor skills) emerge gradually during early life stages instead of being fully present at birth (Harvey and Clutton‐Brock 1985). Thus, after birth, infants depend heavily on maternal or caretaker proximity for comfort, security, and nourishment (Jones 2011). They gradually transition to greater autonomy, with mothers and caretakers reducing physical contact (Maestripieri 1995; Roura‐Torres et al. 2025) and nursing while fostering social interactions (e.g., Pereira and Fairbanks 2002; Pusey 1983; van Noordwijk et al. 2009). Independence typically involves dispersal or integration into existing adult hierarchies, depending on sex and species (Jones 2011). Together, prolonged development and maternal care have been shown to reduce mortality (Zipple et al. 2021) and support the acquisition of competencies needed to survive in complex social environments (Barton and Capellini 2011; Maestripieri and Ross 2004; van Noordwijk and van Schaik 2005). Maternal presence plays a crucial role in protecting infants from aggression and infanticide (e.g., Feldblum et al. 2025), facilitates the development of feeding and locomotor skills through support and scaffolding (Jaeggi et al. 2008; Schuppli et al. 2016), and promotes early social engagement through grooming, play, and proximity (e.g., Maestripieri 2018).

Among primates, humans (*Homo sapiens*) show an extreme pattern of prolonged immaturity, requiring continuous care and support (Hrdy 2001). They reach sexual maturity and adulthood only after an extended period of postnatal development (Bogin and Smith 1996; Leigh 2004). This prolonged development allows for the maturation of the nervous system, facilitating the acquisition of advanced cognitive, social, and cultural skills (Phillips and Shonkoff 2000). This developmental schedule thus imposes extraordinary energetic demands, requiring not only intensive maternal care but extensive alloparental contributions from fathers, grandmothers, and other group members—a cooperative breeding system rare among primates (e.g., Hrdy 2001; Hrdy 2005; Kramer 2010). Additionally, the timing and pacing of key developmental milestones—such as the transition from continuous maternal contact to independent locomotion and spatial exploration—show considerable plasticity across human populations, responding to ecological conditions and social environments (Ellis and Del Giudice 2019; Kuzawa and Bragg 2012). While this extended developmental trajectory distinguishes humans among primates, its evolutionary origins remain difficult to reconstruct directly, as evidence of developmental patterns in extinct hominin ancestors is limited by the fossil record. Informed comparisons with our closest living relatives, the bonobos (*Pan paniscus*) and the chimpanzees (*Pan troglodytes*), therefore offer invaluable insights. Bonobos and chimpanzees share ∼99.6% genetic similarity yet exhibit divergent social ecologies (Prüfer et al. 2012), and humans share some derived traits exclusively with bonobos and others exclusively with chimpanzees—meaning neither species alone represents our ancestral condition (Prüfer et al. 2012). Consistent differences across behavioural and cognitive domains—including social cognition (e.g., Herrmann et al. 2010), tolerance and cooperation (e.g., Hare et al. 2007), communication styles (Fröhlich et al. 2016; Pollick and de Waal 2007), and neural architecture related to empathic sensitivity and prosocial behaviour (Rilling et al. 2012; Stimpson et al. 2016)—have led some to propose that bonobos may be a closer model for particular aspects of human social evolution (e.g., Pisor and Surbeck 2019; Samuni et al. 2022). However, single‐species models are intrinsically limited: they cannot capture the full range of ancestral possibilities or the flexibility inherent in primate development (Gruber and Clay 2016). Variation within and between *Pan* populations is equally important, as behavioural flexibility can be shaped by diverse ecological factors (Gruber and Clay 2016), meaning species‐wide generalizations must be made with caution. Thus, comparisons across both *Pan* species are essential for distinguishing conserved from flexible developmental features, thereby informing hypotheses about the developmental constraints and possibilities that shaped human ontogeny (Kuzawa and Bragg 2012).

Both species share extended juvenile periods with significant maternal support (e.g., Pereira and Fairbanks 2002; Stanton et al. 2020; Surbeck et al. 2011; van Lawick‐Goodall 1967), and live in multi‐male/multi‐female fission‐fusion societies (for reviews, see Brooks et al. 2025; Gruber and Clay 2016). Despite these similarities, bonobos and chimpanzees differ markedly in behavioural traits: bonobos exhibit high levels of adult play and non‐conceptive sexual behaviour, while chimpanzees show cooperative hunting and male‐male alliances (Hare and Yamamoto 2017). Most importantly, their social systems diverge in ways that likely influence developmental trajectories. Bonobos live in predominantly female‐dominated, egalitarian social systems (Furuichi 2011; Kanō 1992; Surbeck and Hohmann 2013, Surbeck et al. 2025), with no reported lethal aggressions (Furuichi 2011; Hohmann 2001; Wilson et al. 2014). Mothers maintain strong lifelong bonds with sons, influencing their reproductive success and social status (Furuichi 1997; Surbeck et al. 2019, 2011). In contrast, chimpanzees live in hierarchical, male‐dominated groups characterized by high levels of inter‐ and intra‐group aggressions, including territorial defence, lethal aggressions (Goodall 1986; Murray et al. 2007; Nishida 2011), and infanticide (e.g., Arcadi and Wrangham 1999; Watts and Mitani 2000; Wilson et al. 2014). These differing social pressures may drive faster behavioural maturation in infant chimpanzees, who may need to develop social and survival skills more rapidly to cope with their competitive environment. In contrast, the safer social conditions of bonobos may facilitate prolonged maternal dependence. For example, chimpanzee juveniles begin facing threats from adult males soon after weaning (approximately 4–5 years), whereas bonobos experience such threats closer to puberty (around the age of 8; Hohmann et al. 2019; Kuroda 1989). However, recent comparative research revealed more nuanced patterns regarding species‐level differences. A recent study found that chimpanzee mothers at Ngogo (Kibale National Park, Uganda) actively supported their offspring, and intervened more frequently during infant attacks than bonobo mothers at Kokolopori (Democratic Republic of Congo, DRC), despite both species' offspring experiencing similar levels of physical harm (Reddy et al. 2024). These findings challenge previous views of bonobo social behaviour, as do other recent studies. For instance, a study using focal data (Altmann 1974)—rather than all‐occurrence sampling—found more frequent male‐male agonistic interactions in one bonobo population (Kokolopori) compared to one chimpanzee population (Gombe, Tanzania; Mouginot et al. 2024). Yet, whether these contrasting social pressures translate into measurable differences in developmental trajectories remains largely unexplored.

Despite these differences, comparative research on *Pan* development is extremely limited (see Table 1). This stems from a long‐standing bias towards chimpanzee studies (Beck 1982)—which benefit from extensive long‐term field sites across tropical Africa (e.g., Goodall 1986; Nakamura et al. 2015; van Lawick‐Goodall 1967; Watts 2012)—and methodological inconsistencies that hinder direct comparisons. In addition, the few available studies on development provided mixed evidence, with Table 1 summarizing the existing research, underscoring variability in research settings and methodologies used. De Lathouwers and Van Elsacker (2006) compared developmental patterns in captive bonobos and chimpanzees and reported differences concerning maternal proximity, grooming and nipple contact. In contrast, Lee and colleagues (2020) focused on female infants and juveniles in wild populations (LuiKotale, DRC, and Gombe, Tanzania) and found no such differences during early development. However, they reported that after the age of 3, female bonobo infants spent more time at greater distances from their mothers compared to female chimpanzee infants. These findings contradict earlier studies suggesting greater maternal dependence in bonobos (De Lathouwers and Van Elsacker 2006; Fröhlich et al. 2016; Koops et al. 2015; Kuroda 1989). However, most studies targeted specific aspects of development rather than investigating full developmental trajectories, and study design differences likely account for such discrepancies. First, captive environments lack natural ecological pressures that shape developmental timing and social behaviour, potentially exaggerating or dampening species differences. Second, the wild study only included female bonobos, whereas the captive study included both sexes, leaving male developmental trajectories in the wild unexplored. It thus remains unclear whether observed differences truly reflect species‐wide patterns or are artefacts of methodological and site‐specific variation. Even within the same site, different methods can yield conflicting results. For instance, Fröhlich and colleagues (2016) relied solely on video recordings of communicative interactions, while Lee and colleagues (2020) applied 1‐min scan sampling across diverse contexts, potentially explaining discrepancies within the same population. Additionally, new methodologies, such as stable isotope analysis, have challenged previous assumptions about weaning processes. While earlier studies based on observations of visible nursing behaviours suggested similar weaning ages between bonobos and chimpanzees (e.g., Kuroda 1989), isotope data now indicate that bonobos seem to wean 2.5–3 years later (Oelze et al. 2024). Within species, weaning age also varies across populations (e.g., Lonsdorf et al. 2020), underscoring the role of ecological and demographic factors on behavioural output. These complexities highlight the need for caution and use of data across populations and species to tease apart whether differences reflect population or species differences.

**TABLE 1:** Overview of current research comparing bonobo and chimpanzee development across behavioural aspects, species, location (wild or captive), and sex (if known).

Aspect	Bonobos	Location	Sex	Chimpanzees	Location	Sex
**Weaning**	Weaning at 4 to 5 years^1^; up to 7 years^9^	Wamba^1^, LuiKotale^9^	Unspecified	Weaning at 4 to 5 years^10,11^; up to 7 years^11^	Ngogo^10,11^, Kanywara^11^, Mahale^11^, Gombe^11^	Both
**Social proximity**	Never leave mother (3 months)^1^	Wamba^1^	Unspecified	Never leave mother (3–5 months)^2,3^	Gombe^2^, Taï^3^	Both
	< 1 m away at 6 months^1^	Wamba^1^	Unspecified			
	Explore, touch siblings and unrelated individuals at 10 months^1^	Wamba^1^	Unspecified	Parting from mothers, and approach unrelated individuals at 6 months^1^	Unclear^1^	Unspecified
	> 10 m at 3 years (but never too far to return immediately)^1^	Wamba^1^	Unspecified	Spending time out of maternal proximity at 2.5–3 years^1^	Unclear^1^	Unspecified
	Spend 25% of time > 5 m from mother (4–5 years), increasing to 50% at 5–8 years^6^	LuiKotale^6^	Females	Spending 25% of time > 5 m from mother (5–8 years)^6^	Gombe^6^	Females
	Spend more time in close proximity to mother compared to chimpanzees^4,7,8^	LuiKotale^7^, Wamba^7,8^, Captivity ^4^	Both	Spending more time at a greater distance from mother, compared to bonobos^4^	Captivity ^4^, Taï^7^, Kanywara^7^, Kalinzu^8^	Both
	Maternal contact (50% of the time) from 1–2 years^6^	LuiKotale^6^	Females	Maternal contact (50% of the time) from 1 to 2 years^6^	Gombe^6^	Females
**Locomotion**	Ride mothers until 6 years (50% of the time until 4–5 years), no difference between ventral or dorsal^6^	LuiKotale^6^	Females	Riding on mothers until 4–5 years (50% of the time until 3 years), no difference between ventral or dorsal^6^. Gombe ends around 2.5 years^2^	Gombe^2,6^	Both
	Ventral clinging position in most cases (80%) at 2 years^1^	Wamba^1^	Unspecified	Ventral riding during the first 6–9 months^2^, shifts by 2 years^5^	Gombe^2,5^	Both
	Dorsal riding becomes more common from 3 years onwards, especially during long‐distance travel^1^	Wamba^1^	Unspecified	Dorsal riding starts at around 7 months^2,3^, males transition earlier^5^; ends at 4–4.5 years^5^	Gombe^2,5^, Taï^3^	Both
				Independent travel begins around 3 years, males started earlier^5^	Gombe^5^	Both
**Feeding**	Trying solid food, but not eating it < 1 year^1^	Wamba^1^	Unspecified	Eating of solid food starts around 6 months^3^	Taï^3^	Both
	More nipple contact than chimpanzees from 3 years onwards^4^	Captivity ^4^	Both	Continuous suckling during infancy^2^, Less nipple contact than bonobos from 3 years onwards^4^	Gombe^2^, Captivity^4^	Both
	Suckling continues until 5–6 years^6^	LuiKotale^6^	Females	Suckling continues until 5–6 years^6^	Gombe^6^	Females
**Grooming patterns**	Social grooming observed from 2–3 years^6^, and more time grooming other group members compared to chimpanzees^4^	LuiKotale^6^, Captivity^4^	Females	Social grooming starts around 1.5 years in Taï^3^ and from 2–3 years in Gombe^6^; and spend less time grooming other group members compared to bonobos^4^	Gombe^6^, Taï^3^, Captivity^4^	Both
				Mother–infant grooming observed rarely in the first few weeks, frequency increases with age^2^	Gombe^2^	Both

*Source:* (1) Kuroda (1989), (2) van Lawick‐Goodall (1967), (3) Bründl et al. (2021), (4) De Lathouwers and Van Elsacker (2006), (5) Lonsdorf et al. (2014), (6) Lee et al. (2020), (7) Fröhlich et al. (2016), (8) Koops et al. (2015), (9) Oelze et al. (2024), (10) Bădescu et al. (2017), (11) Lonsdorf et al. (2020).

Data collection methods: Observational, no described method^1,2^; Focal follows^3,4,5,6,8,11^ (all day^3,11^; several hours—full day (chimps) and 1 h (bonobo)^6^; 30–120 min^8^); instantaneous scan sampling^3,4,5,6,8^ (every 15 s^4^; 1 min^3,5,6^; 2 min^8^); video recordings of focal infants^7^; faecal isotope analyses^9,10^.

Locations: Captivity (7 zoos across Europe); Gombe (Tanzania); Kalinzu (Uganda); Kanyawara (Uganda); LuiKotale (Democratic Republic of the Congo); Mahale (Tanzania); Ngogo (Uganda); Taï (Côte d'Ivoire); Wamba (Democratic Republic of the Congo).

Beyond species‐level factors, individual and dyadic variation may also shape developmental trajectories. Multiparous mothers (females with prior offspring) may provide more effective care than primiparous (first‐time) mothers due to experience or energetic trade‐offs (Amici et al. 2025; Fairbanks 1996; Stanton et al. 2014). The presence of younger siblings can also create competition for maternal resources (Devinney et al. 2001; Oelze et al. 2024; Shivani et al. 2025), especially when lactation overlaps with gestation, potentially accelerating infant independence. Offspring sex may also influence maternal investment due to sex‐biased dispersal and fitness returns, especially in male‐philopatric species—like chimpanzees and bonobos—where mothers may invest more heavily in sons who remain and yield long‐term benefits (Lonsdorf et al. 2014; Nakamichi 1989). Accounting for such variation is essential for understanding the full range of factors that shape early development in great apes.

### 1.1 Aim and Hypotheses

This study investigated early behavioural development in two wild bonobo and chimpanzee populations, employing consistent, quantitative methodologies. We focused on how species‐specific social dynamics and individual‐level factors—such as maternal experience, sibling presence, and infant sex—may shape the pace and nature of developmental trajectories. To do so, we examined the following research questions:

1. **Do bonobo and chimpanzee infants differ in the timing of behavioural markers of independence?** To address this question, we assessed the occurrence of specific behaviours across development that reflect changing levels of maternal dependence in travel (ventral riding, dorsal riding, independent travel), feeding (nipple contact), and grooming (grooming directed towards the mother). If species‐specific social structure and risk exposure place different developmental demands on infants, we expected chimpanzee infants to develop locomotor, nutritional and social competencies earlier than bonobo infants. Alternatively, if these developmental patterns are relatively conserved across species, we expected similar timelines of behavioural change in the two species.
2. **Do bonobo and chimpanzee infants differ in the timing of spatial independence from their mothers?** To examine this question, we assessed the frequency of spatial proximities (< 1, 1–5, > 5 m) between infants and their mothers across development. If social pressures such as predation risk, male aggression, or group cohesion dynamics influence mother–infant distances, we expected chimpanzee infants to remain in closer proximity to their mothers for longer periods compared to bonobo infants. Alternatively, if spatial independence develops similarly regardless of species‐specific social pressures, we expected comparable developmental trajectories in mother–infant proximity in both species.

## 2 Methods

### 2.1 Study Communities

We conducted our study at the Kokolopori Bonobo Reserve, DRC (April to November 2022), and the Ngogo Chimpanzee Project, Kibale National Park, Uganda (February–September 2021 and August 2022–February 2023). Five fully habituated communities were observed: three bonobo communities (Fekako, FKK; Ekalakala, EKK; and Kokoalongo, KKL) of the Kokolopori population, and two chimpanzee communities (West and Central) of the Ngogo population. The bonobos have been monitored since 2007 through a collaboration between NGOs Vie Sauvage and Bonobo Conservation Initiative, with M.S. initiating long‐term detailed research on this population in 2016 (Surbeck et al. 2017). The chimpanzees have been studied since 1995 (Wood et al. 2017), allowing individual identification and continuous long‐term data collection in both populations. For this study, we focused on infants aged 0–5.5 years (*N* = 43; 21 males, 22 females; see Supplementary Materials S1 for full details).

### 2.2 Behavioural Observations

#### 2.2.1 Bonobo Data Collection

Data were collected by J.V. (April‐November 2022) and F.W. (April–October 2022), for their respective projects, using standardized methodologies later combined for this comparative study (see Table 2). J.V. conducted continuous focal animal sampling (Altmann 1974; Martin and Bateson 1993) during 2‐h focal follows using Cybertracker software (v3.520) on a smartphone (Cyrus CS24) to record general behavioural data on development (see behavioural parameters below). Information such as the start and end of each focal follow, and periods when the focal animal was out of sight were also recorded. This method was complemented with instantaneous scan sampling (Altmann 1974; Martin and Bateson 1993) at 15‐min intervals, recording specific behaviours of the focal individual, the mother, and proximity to other individuals and the mother. F.W. also conducted scan sampling at 15‐min intervals during 1‐h follows using voice notes. J.V. and F.W. maintained a record of the total duration for which a focal had been observed per month and gave priority to those who had been sampled less in cases where multiple focal individuals were present. Following each focal follow, a new focal subject was chosen, given consistent grouping of bonobos. Concurrent observations were occasionally conducted, with efforts made to observe different focal subjects to minimize overlap and with overlapping scans being excluded from analyses.

Party composition data were collected every 30 min by local field assistants, cumulatively aggregating the presence of each individual observed within the past 30 min. Bonobos were continuously followed from when they emerged from their nests (05:30–06:00) until approximately 15:00 daily, resulting in a total of 222 observation hours (FKK = 28; EKK = 67; KKL = 127). To capture developmental changes over time, each focal infant was followed for approximately 1 h per month (average = 66 ± 34 min/infant/month), except for infants under 1 year of age, who were followed for 30 min per month (average = 34 ± 19.5 min/infant/month). F.W. followed each infant for about 30 min per month (average = 37 ± 14 min/infant/month), regardless of age. To compare behavioural development, the data of all infants (*N* = 21), except one orphaned individual, were included in the subsequent analyses.

#### 2.2.2 Chimpanzee Data Collection

Data were collected by B.v.B. (February–September 2021; August 2022–February 2023) and R.K. (October 2022–February 2023) following a protocol similar to J.V.’s bonobo data collection (continuous focal animal sampling complemented with 15‐min instantaneous scan sampling; see Table 2), adapted for chimpanzee observation conditions. Unlike bonobo observations, B.v.B. and R.K. chimpanzee focal follows were conducted for full days (8:00–16:00) due to better visibility of the chimpanzees, enabling the observation of one focal individual per day. Party composition data were recorded cumulatively every hour. Concurrent observations never occurred. In total, 1505 h of observations were collected (West = 1105, Central = 400). Each infant was followed approximately 6 h per month (> 1 year: average = 461 ± 203 min/infant/month; < 1 year: 555 ± 249 min/infant/month). To compare behavioural development, infants were included in the analysis if sufficient data had already been collected in previous months, allowing for more reliable developmental tracking.

**TABLE 2:** Summary of data collected by observer, species, and community, sampling methodology, duration, and total observation time.

Observer	Species and community	Sampling methods	Duration	Total observation time (hours)
J.V.	Bonobos (FKK, EKK, KKL)	Focal animal (continuous)	April–November 2022	166
		Instantaneous scan (15 min intervals)		
F.W.	Bonobos (FKK, EKK, KKL)	Instantaneous scan (15 min intervals)	April–October 2022	56
B.v.B.	Chimpanzees (West, Central)	Focal animal (continuous)	February–September 2021 and August 2022–February 2023	1244
		Instantaneous scan (15 min intervals)		
R.K.	Chimpanzees (West)	Focal animal (continuous)	October 2022–February 2023	261
		Instantaneous scan (15 min intervals)		

*Note*: Focal and scan data were analysed separately to address different research questions (Question 1: behavioural markers of independence; Question 2: spatial independence).

Abbreviations: B.v.B., Bas van Boekholt; F.W., Franziska Wegdell; J.V., Jolinde M.R. Vlaeyen; R.K., Raymond Katumba.

### 2.3 Description of Parameters

Following established ethograms (e.g., Goodall 1986; Lonsdorf et al. 2014; Nishida et al. 1999), we utilized the proxies described below to measure (1) behavioural markers of independence and (2) spatial independence from mothers (Table 3). Behavioural data were collected via focal animal sampling, while proximity data were obtained through scan sampling (Altmann 1974; Martin and Bateson 1993). As our primary aim was to chart developmental trajectories from the infant's perspective, we specifically focused on infant behaviours rather than maternal behaviours.

**TABLE 3:** Summary of data collected for each research question and definitions of behavioural categories.

	Category	Behaviour	Definition
**Focal data**	Travel *(locomotor development)*	*Ventral*	Infant rides ventrally, clinging to the mother's belly using hands and feet; the mother may provide additional support with one hand.
		*Dorsal*	Infant rides dorsally, lying or sitting on the mother's back during transport.
		*Independent*	Infant moves on its own (quadrupedally) for at least 5 m, possibly still in contact with the mother.
	Feeding *(nutritional development)*	*Nipple contact*	Infant takes a nipple in the mouth, indicating ongoing reliance on maternal milk.
	Grooming *(social development)*	*Grooming (infant to mother)*	Infant grooms the mother by parting her hair and picking at exposed skin with its hands.
**Scan data**	Spatial independence	*< 1 m*	Infant is within arm's reach of the mother (body contact possible).
		*1–5 m*	Infant is between 1 and 5 m from the mother.
		*> 5 m*	Infant is more than 5 m away from the mother.

### 2.4 Statistical Analyses

#### 2.4.1 Inter‐Rater Reliability

Since the cooperation project began after data collection was completed, an inter‐rater reliability test could not be conducted for the bonobo dataset. However, J.V. and F.W. discussed all definitions of their scan data in great detail to ensure consistency and comparability of collected data and data analysis. For the chimpanzee dataset, inter‐rater reliability was assessed between B.v.B. and R.K. for both scan and focal data. Cohen's Kappa values for scan data indicated substantial overall agreement (*κ* = 0.697; Landis and Koch 1977), with *κ* = 0.697 (< 1 m), *κ* = 0.406 (1–5 m), and *κ* = 0.827 (> 5 m). Focal data comparisons across observers also showed high agreement for most behaviours (travel, nursing, grooming; see Supplementary Materials S2 for details).

#### 2.4.2 Statistical Models

To compare developmental trajectories between bonobo and chimpanzee infants, we ran two main models. To answer question one, we used similar models per behavioural category to assess how different behavioural aspects varied with age and between species (*Locomotion*: Model 1a‐c; *Feeding*: Model 2; and *Grooming*: Model 3). To answer question two, we applied a model testing how mother–infant proximities change with increasing age (Model 4). All models were fitted in R (version 2025.09.0; R Core Team 2025), using the ‘glmer’ function of the *lme4* package (version 1.1.37; Bates et al. 2015) for Models 1 to 3, and the ‘clmm’ function of the *ordinal* package for Model 4 (version 2023.12‐4.1; Christensen 2019). We determined collinearity using the ‘vif’ function of the *car* package (Fox and Weisberg 2019). Details about model assumptions and stability tests can be found in the Supplementary Materials S2.

#### 2.4.3 Model Specificities

To maintain methodological consistency and ensure valid comparisons, focal sampling data were used exclusively for Research Question 1 (behavioural markers of independence), while scan sampling data were used exclusively for Research Question 2 (spatial independence). The two data types were not combined in any statistical model.

##### 2.4.3.1 Question One—'Do Bonobo and Chimpanzee Infants Differ in the Timing of Behavioural Markers of Independence?'

To analyse age‐ and species‐based differences in travel, feeding, and grooming behaviours, we applied generalized linear mixed models (GLMM; Baayen 2008) per behaviour: travel modes (*Ventral riding—*Model 1a, *Dorsal riding—*Model 1b, *Independent travel*—Model 1c), feeding modes (*Nipple Contact—*Model 2), and grooming (Model 3). For Models 1 and 2, a binomial distribution was fitted with behaviour frequency as the response variable, represented by a two‐column matrix of successes and failures per individual per observed day (Baayen 2008). The definition of success and failure depended on the specific behaviour analysed: for travel, the behaviour of interest (ventral, dorsal, or independent travel) was coded as a success, while the other two travel modes were considered failures; for feeding, nipple contact was treated as a success, with independent feeding classified as a failure. For Model 3, a binomial distribution was fitted with infant‐mother grooming occurrences per individual per observed day (Yes, No) as the response variable.

Throughout all models, fixed effects were centred and included age (*z*‐transformed), species (Bonobo, Chimpanzee), sex (F, M), presence of a younger sibling (whether the infant at the observation age had a younger sibling; Yes, No), and mother parity (Primiparous, Multiparous). For *Dorsal riding*, a quadratic age term was added due to non‐linear patterns. A three‐way interaction (age, species, sex) and a two‐way interaction (age, sibling presence) were tested and excluded if non‐significant. Random intercepts included individual, mother (accounting for multiple individuals with the same mother), community (five levels), date and individual nested in date (accounting for multiple individuals observed on the same day). To maintain precision in fixed effects estimates and keep the Type I error rate at 5%, we included all theoretically identifiable random slopes (e.g., age within individual and mother, and age, sex, and sibling presence within community) but excluded correlations among intercepts and slopes (Schielzeth and Forstmeier 2009). Model fit was compared to null models, comprising only sibling presence and mother parity, using likelihood ratio tests. Collinearity was low (maximum VIF_Model1a_: 1.08; VIF_Model1b_: 2.1; VIF_Model1c_: 1.8; VIF_Model2_: 1.97; VIF_Model3_: 1.28). The sample analysed included 563 observations (42 individuals) for Model 1, 516 observations (43 individuals) for Model 2, and 463 observations (43 individuals) for Model 3.

##### 2.4.3.2 Question Two—'Do Bonobo and Chimpanzee Infants Differ in the Timing of Spatial Independence From Their Mothers?'

To examine whether mother–infant proximities varied with regard to age and species, we fitted an ordinal mixed model (cumulative logit link; Agresti and Kateri 2017) using the measured distances to mothers (three levels: < 1 m, 1–5 m, > 5 m) as the response variable. Fixed effects were centred and included age (*z*‐transformed), species, sex, presence of a younger sibling and mother parity. Additionally, the number of scans per individual was included as an offset term to account for differences in observation effort and ensure comparability across individuals. A three‐way interaction (age, species, sex) was tested, but we excluded it due to being non‐significant. Random intercepts included individual, mother, community (five levels), date, and individual nested within date (accounting for multiple individuals observed on the same day). We also included the number of individuals and siblings within 5 m to control for potential alternative interactants (we excluded the number of males within 5 m and the number of females within 5 m as these were correlated with the number of individuals within 5 m). Party size and daily rainfall were also included as predictors. Party size was included to account for potential influences on mother–infant proximity, as these may vary in large versus small groups, and rainfall was included as proximity decreases during rain (Vlaeyen and van Boekholt, personal observation). To maintain precision and control the Type I error rate (5%), we included theoretically identifiable random slopes (e.g., age within individual and mother, and age, sex, and sibling presence within community) but excluded correlations among intercepts and slopes (Schielzeth and Forstmeier 2009). Model significance was assessed by comparing the full model to a null model, comprising only sibling presence, using a likelihood ratio test. Collinearity was negligible (maximum VIF: 1.1). The dataset included 5,004 observations for 43 individuals.

## 3 Results

### 3.1 Question One—Behavioural Markers of Independence

#### 3.1.1 Model 1a—Ventral Riding

**FIGURE 1:**
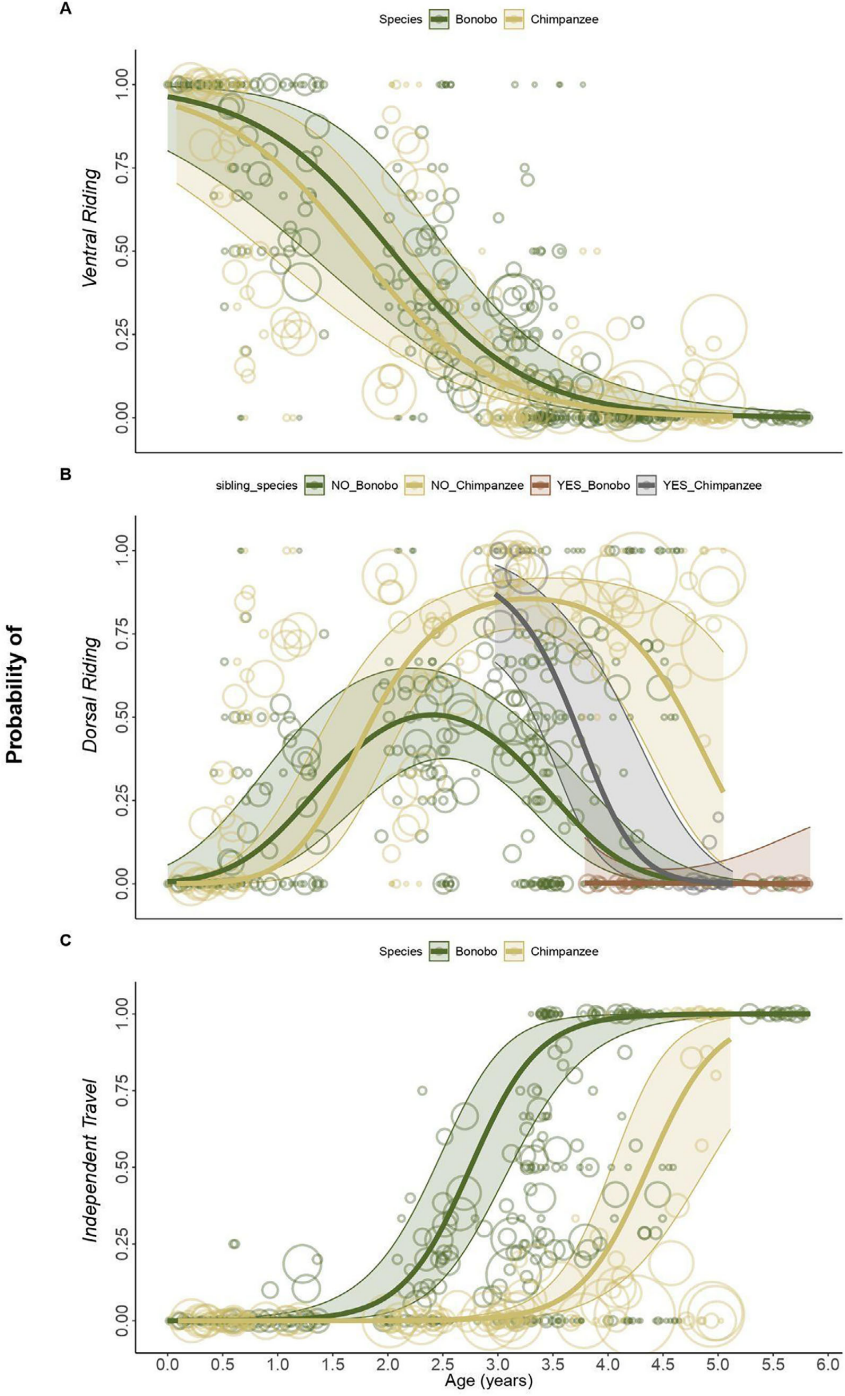
Developmental trajectories of infant locomotion. Probability plots for bonobo (dark green) and chimpanzee (yellow) infants (A) riding ventrally, (B) riding dorsally, and (C) travelling independently (the opposite of riding on mother) as a function of age (in years), including the 95% asymptotic confidence intervals. Bubbles depict the raw data, with bubble area being proportional to the number of observations for each infant/age combination (range 1–60), scaled for better visibility. For panel (B), we depict the results of a different model combining species and presence of younger siblings (YES/NO) to show both statistical effects in one figure.

In both bonobos and chimpanzees, ventral riding declined with increasing age (*β* = –2.57, SE = 0.49, *z* = –5.27, *p* < 0.001, 95% CI [–3.52, –1.62]; Figure 1A), with no significant difference between species (*β* = –0.49, SE = 0.38, *z* = –1.29, *p* = 0.192, 95% CI [–1.24, 0.25]). Maternal parity had a significant effect across species (*β* = 0.98, SE = 0.43, *z* = 2.27, *p* = 0.021, 95% CI [0.15, 1.82]), with infants of primiparous mothers showing higher rates of ventral travel. Other factors, such as sex and sibling presence, had no significant effects. Overall, the test predictors had a clear effect on the model (full vs. null model: *χ^2^* (3) = 15.9, *p* = 0.001).

#### 3.1.2 Model 1b—Dorsal Riding

For both species, dorsal riding increased with age to a peak, before declining as age increased (age^2^: *β* = –2.18, SE = 0.34, *z* = –6.40, *p* < 0.001, 95% CI [–2.85, –1.51]; Figure 1B). Chimpanzee infants exhibited extended dorsal riding compared to bonobos, as indicated by a significant interaction between age and species (*β* = 2.43, SE = 0.69, *z* = 3.52, *p* < 0.001, 95% CI [1.08, 3.78]; Figure 1B). For both species, infants of multiparous mothers exhibited higher dorsal riding rates compared to infants of primiparous mothers (*β* = –1.07, *SE* = 0.38, *z* = –2.78, *p* = 0.005, 95% CI [–1.82, –0.31]), and infants without younger siblings rode dorsally longer on their mothers, as indicated by a negative age‐sibling presence interaction (*β* = –4.16, *SE* = 1.74, *z* = –2.39, *p* = 0.017, 95% CI [–7.57, –0.75]; Figure 1B). Sex had no effect. Overall, the test predictors had a clear effect on the model (full vs. null model: *χ^2^* (7) = 69.61, *p* < 0.001).

#### 3.1.3 Model 1c—Independent Travel

In both species, independent travel increased significantly as infants grew older (*β* = 5.17, SE = 0.78, *z* = 6.62, *p* < 0.001, 95% CI [3.64, 6.70]; Figure 1C). However, bonobo infants travelled independently more frequently than chimpanzee infants, regardless of age (species effect: *β* = –5.09, SE = 0.75, z = –6.75, *p* < 0.001, 95% CI [–6.57, –3.61]; Figure 1C). For both species, infants with a younger sibling also travelled independently more frequently than those without a younger sibling (*β* = 2.53, SE = 0.78, *z* = 3.24, *p* = 0.001, 95% CI [1.00, 4.06]). Neither sex nor maternal parity had a significant effect on the test parameters. Since this model examined the inverse of riding on the mother, it also showed that chimpanzee infants travelled more often on their mothers—regardless of age—and infants without younger siblings did so for longer durations. Overall, the test predictors had a clear effect on the model (full vs. null model: *χ^2^* (3) = 29.33, *p* < 0.001).

#### 3.1.4 Model 2—Nipple Contact

**FIGURE 2:**
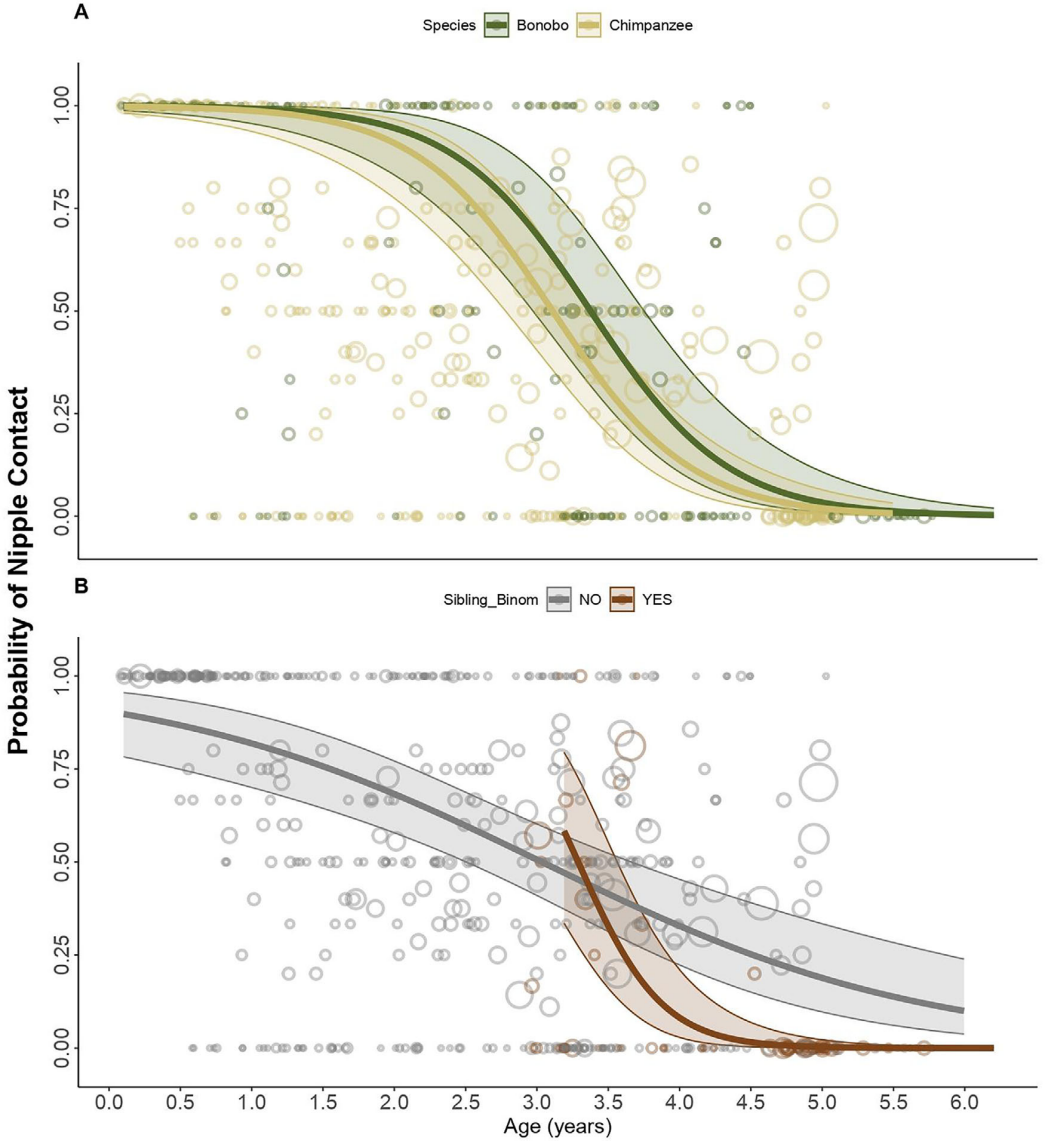
Developmental trajectories of infant nipple contact. Probability plots for bonobo (dark green) and chimpanzee (yellow) infants' nipple contact (the opposite of independent feeding) as a function of age (in years), including the 95% asymptotic confidence intervals. Bubbles depict the raw data, with bubble area being proportional to the number of observations for each infant/age combination (range 1–21), scaled for better visibility.

In both species, nipple contact decreased significantly with increasing infant age (*β* = –1.16, SE = 0.24, *z* = –4.84, *p* < 0.001, 95% CI [–1.63, –0.69]; Figure 2), with no significant species difference (*β* = –0.55, SE = 0.35, *p* = 0.120, 95% CI [–1.25, 0.14]). Infants with a younger sibling showed an earlier decline in nipple contact over age, as indicated by a significant age‐sibling presence interaction (*β* = –4.16, SE = 0.96, *z* = –4.34, *p* < 0.001, 95% CI [–6.04, –2.28]). Sex and mother parity had no significant effects on nipple contact. Since this model examined the inverse of independent feeding, these results also indicate that infants with younger siblings began feeding independently earlier than those without. Overall, the test predictors had a clear effect on the model (full vs. null model: *χ^2^* (4) = 33.3, *p* < 0.001).

#### 3.1.5 Model 3—Grooming

**FIGURE 3:**
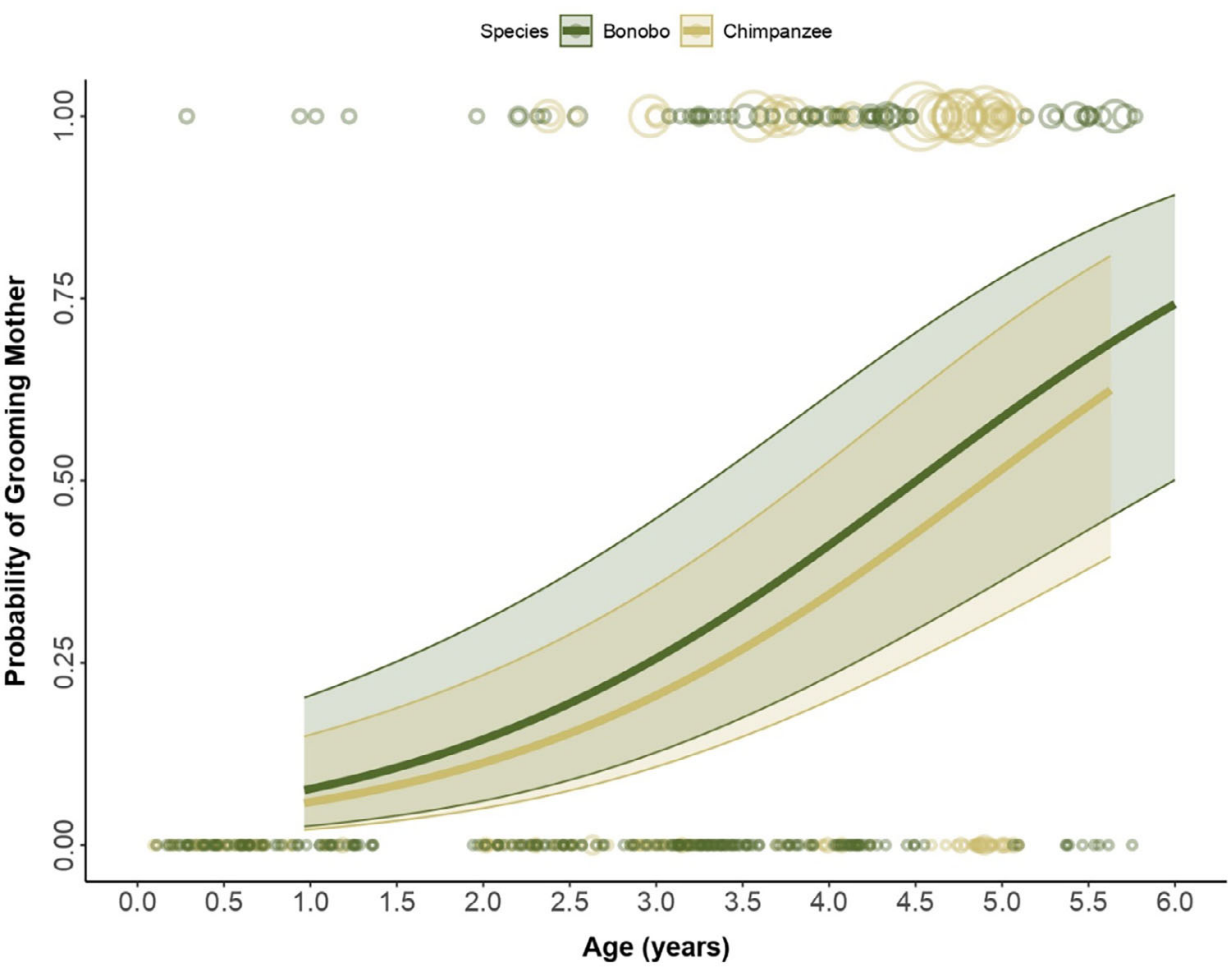
Developmental trajectories of infants grooming their mothers. Probability of bonobo (dark green) and chimpanzee (yellow) infants grooming their mothers as a function of age (in years), including the 95% asymptotic confidence intervals. Bubbles depict the raw data, with bubble area being proportional to the number of observations for each infant/age (range 0–13) combination, scaled for better visibility.

In both species, infant grooming of their mothers increased significantly with age (*β* = 1.11, SE = 0.22, *z* = 4.96, *p* < 0.001, 95% CI [0.67, 1.54]; Figure 3), with no significant species difference (*β* = –0.29, SE = 0.40, *p* = 0.470, 95% CI [–1.07, 0.49]). Other predictors, including sex, sibling presence, and mother parity, had no significant effect. Overall, the test predictors showed a clear effect on the model (full vs. null model: *χ^2^* (3) = 11.02, *p* = 0.011).

While the travel and nursing models revealed some individual variation in developmental trajectories, these patterns were robust overall, with age effects consistently emerging across individuals. Full model outputs and additional descriptive data can be found in Supplementary Materials S3.

### 3.2 Question Two—Spatial Independence Patterns

#### 3.2.1 Model 4—Spatial Independence

**FIGURE 4:**
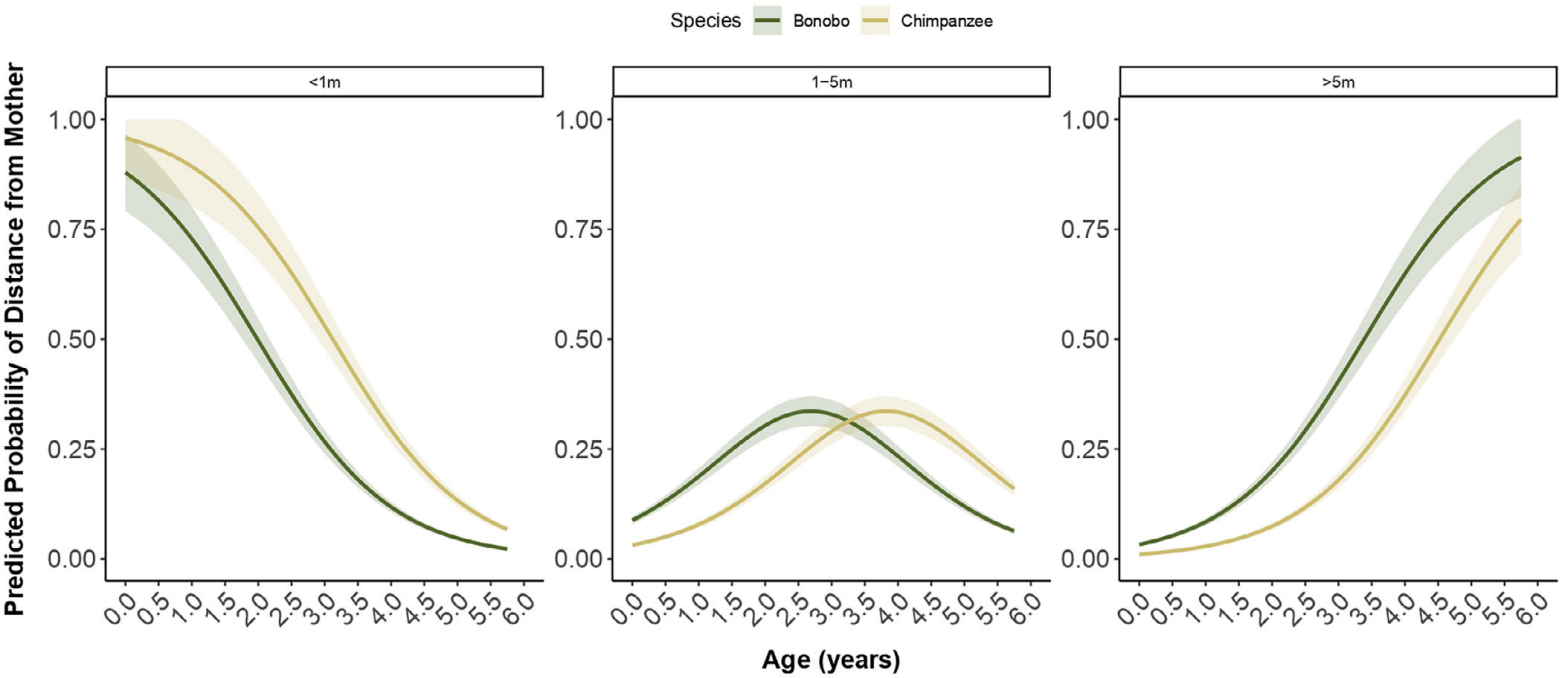
Developmental trajectories of infant spatial proximities from their mothers. Predicted probabilities of an infant's distance from its mother at different ages (in years) in bonobos (dark green) and chimpanzees (yellow), including the 95% asymptotic confidence intervals.

In both species, older infants were observed farther from their mothers than younger ones, as indicated by a significant positive effect of age on distance to mother (*β* = 1.46, SE = 0.14, z = 10.53, *p* < 0.001, 95% CI [1.19, 1.74]; Figure 4). Species differences were observed (*β* = –1.13, SE = 0.23, *z* = –4.83, *p* < 0.001, 95% CI [–1.59, –0.67]; Figure 4), with infant bonobos being observed less frequently in the < 1 m category, and more frequently in the > 5 m category compared to chimpanzee infants. Specifically, chimpanzee infants were 68% less likely to transition to a more distant proximity category compared to bonobos (odds ratio = 0.32), suggesting they remained closer to their mothers for longer. However, there was no significant interaction between age and species, suggesting that both species transition at a similar rate, but at different ages. Other factors, including sex, sibling presence, or mother parity, had no significant effect (see Supplementary Materials S3). The model's steep threshold coefficients (threshold 1: *β* = 5.02, SE = 0.34, 95% CI [4.36, 5.69]; threshold 2: *β* = 6.42, SE = 0.34, 95% CI [5.75, 7.09]) reflect a strong and consistent shift towards greater spatial independence with increasing age. Overall, the model was highly significant (full vs. null model: *χ^2^* (3) = 64.12, *p* < 0.001).

Individual differences in the pace of spatial independence were observed, yet the model still captured clear age‐ and species‐related trends. Full model outputs and additional descriptive data can be found in Supplementary Materials S3.

## 4 Discussion

This study investigated developmental trajectories in wild bonobos and chimpanzees across two populations, examining how species‐specific social dynamics and individual factors—maternal experience, sibling presence, and infant sex—influence travelling, feeding, grooming, and spatial independence. Age was a significant predictor across all tested parameters, validating these behaviours as robust developmental measures. While bonobo and chimpanzee infants showed similar trajectories for ventral riding, nursing, and grooming, they differed in dorsal riding, independent travel, and spatial independence. Chimpanzees rode dorsally longer than bonobos of the same age, and stayed closer to their mothers longer, while bonobos travelled independently more frequently at younger ages. Beyond species differences, maternal parity and sibling presence influenced development in both species. Infants of primiparous mothers showed higher rates of ventral travel, while infants of multiparous mothers exhibited higher rates of dorsal riding. Infants without younger siblings rode dorsally longer, travelled independently less frequently, and nursed longer compared to those with younger siblings. Sex showed no effect on any investigated behavioural parameters. The following paragraphs discuss these findings and their broader implications for development and primate evolution in more detail.

### 4.1 Development of Travelling

We identified several species‐level differences between both populations regarding dorsal and independent travel. Regardless of age, bonobo infants travelled independently more compared to chimpanzee infants. These results may reflect species differences in locomotor behaviour. For instance, a study on adult bonobos and chimpanzees at Lomako, DRC, and Taï, Côte D'Ivoire, showed that bonobos were more arboreal than chimpanzees (Doran 1993). If bonobo mothers spend more time in trees, where carrying infants is more challenging due to the need to navigate branches and maintain balance, this may encourage earlier independent locomotion in bonobo infants compared to chimpanzee infants whose mothers travel more terrestrially. Although we lack comparable chimpanzee data, bonobo mothers in our study carried infants more often on the ground (60.5%) than in trees (39.5%), while independent travel was more common in trees (77%) compared to the ground (23%; see Supplementary Materials S4). These differences may also result from habitat differences. The closed‐canopy primary and heterogeneous forests of Kokolopori (e.g., Lucchesi et al. 2021; Surbeck et al. 2017) likely facilitate arboreal travel, whereas Ngogo's mixed landscape of colonizing and old‐growth forests with grasslands (Wing and Buss 1970) may promote terrestrial travel, and thus greater reliance on maternal carrying. Another possibility is that travel differences reflect social systems, with chimpanzees facing higher risks of lethal aggression and infanticide (Arcadi and Wrangham 1999; Lowe et al. 2020; Watts and Mitani 2000). Chimpanzees may thus adopt protective strategies such as prolonged carrying, as reflected by higher maternal intervention rates in offspring conflicts, compared to bonobos (Reddy et al. 2024). These differences echo protective caregiving strategies observed in certain human hunter‐gatherer societies (e.g., the !Kung San, Ache of Paraguay, Agta of the Philippines), where harsher environments necessitate extended close proximity to caregivers to enhance survival (e.g., Konner 2017).

We also found a species‐by‐age difference regarding dorsal riding, with chimpanzee infants showing higher rates of dorsal riding compared to age‐matched bonobos, particularly at older ages. This contrasts with findings by Lee and colleagues (2020) at LuiKotale, DRC, and Gombe, Tanzania, who reported the opposite pattern. Differences between these studies may reflect population‐specific ecological and social factors. At LuiKotale, bonobo mothers often carried two dependent offspring simultaneously—an occurrence rare among chimpanzees at Gombe (Lee et al. 2020). In contrast, chimpanzee mothers in our study carried their infants both ventrally and dorsally concurrently, whereas such occurrences were rarely observed among bonobos. Only three instances were noted: one post data collection (Lily Fornof, personal communication), and two involving the same mother—one during a stressful event involving a loose dog in the forest (Vlaeyen and Wegdell, personal observation). Older bonobo infants occasionally attempted bipedal travel while holding onto their mothers’ backs. Conflicts frequently arose when infants tried to ride dorsally, particularly in the presence of a new sibling (Vlaeyen, personal observation), suggesting mothers may actively regulate travel strategies during pregnancy or when managing multiple offspring.

Furthermore, we found high similarity between species in how maternal experience and sibling presence shaped infant travel. Infants without younger siblings rode dorsally longer and travelled independently less frequently compared to those with younger siblings. For bonobos, this pattern mirrors findings from LuiKotale, where sibling births triggered significant reductions in maternal carrying (Behringer et al. 2022). Such shifts likely reflect maternal strategies to balance the energetic demands of supporting multiple offspring while simultaneously fostering independence in older infants (Emery Thompson et al. 2016; Hrdy 2001; Revathe et al. 2024). Additionally, infants of primiparous mothers engaged in more ventral riding, whereas infants of multiparous mothers spent more time riding dorsally. This distinction may reflect variations in maternal experience. First‐time mothers, who are often less skilled, more protective and less rejective (Fairbanks 1996; Simpson et al. 1981), may favour ventral riding for enhanced safety, while experienced mothers may encourage older infants to ride dorsally, promoting gradual independence. These findings align with other studies, including those on humans, showing that first‐time mothers tend to invest more in their firstborn (e.g., Hertwig et al. 2002; Simpson et al. 1981; Stanton et al. 2014).

Our findings on infant travel behaviour contrast with previous research suggesting slower behavioural development in bonobos (Kuroda 1989; Lee et al. 2020), instead revealing population‐level variation in maternal styles. Mothers from both species adjusted their travel strategies based on offspring age, maternal experience, and sibling presence. Although cross‐cultural data remain limited and caregiving strategies vary, some hunter‐gatherer societies (e.g., the !Kung San, Ache of Paraguay, Agta of the Philippines) exhibit similar developmental patterns. Early infancy is characterized by near‐continuous maternal carrying, with close proximity maintained through slings or body wraps, transitioning to clinging to maternal baskets, followed by piggyback rides from caregivers by age 3 to 5, before children are encouraged to walk independently after the age of 5 (Hewlett et al. 1998; Konner 2017). These shifts reflect maternal strategies to balance caregiving with fostering independence, a pattern mirrored in our findings where sibling presence accelerates transitions to independent travel (Barnett et al. 2022).

### 4.2 Development of Nipple Contact

Our findings revealed no significant differences between species, as nipple contact persisted for extended periods, but sibling presence seemed to accelerate the weaning process. Infants with younger siblings showed steeper declines in nipple contact with age compared to those without. Our findings align with research across primates, demonstrating that sibling birth plays a key role in shaping weaning patterns. For instance, a study of wild rhesus macaques (*Macaca mulatta*) found that infants who experienced weaning completion at the time of sibling birth exhibited heightened distress behaviours (Devinney et al. 2001). In contrast, weaning‐related behaviours in bonobos decline gradually before sibling birth rather than ceasing abruptly (Behringer et al. 2022). This suggests that mothers may gradually reduce nursing opportunities in relation to reconception and upcoming sibling arrival. It is worth noting that nipple contact may persist beyond nutritional weaning, as some infants engage in comfort nursing without milk intake (e.g., Bădescu et al. 2017). A similar pattern occurs in human hunter‐gatherer societies, where nipple contact often continues for up to 4 years but typically declines and ends during the mother's next pregnancy, often before the birth of the next child (Konner 2017). Although weaning practices vary considerably across hunter‐gatherer groups, in the absence of a younger sibling, breastfeeding may extend to the age of 5 (Konner 2017), whereas in W.E.I.R.D societies, weaning tends to occur earlier due to cultural and practical constraints (e.g., Dettwyler 2004; Runjić Babić 2020). Our findings contribute to evidence of prolonged maternal investment in *Pan*, consistent with the gradual weaning patterns that characterize most mammalian species (Hinde and Milligan 2011; Langer 2008).

### 4.3 Development of Grooming

While prior work has predominantly focused on adult individuals in both chimpanzees (e.g., Girard‐Buttoz et al. 2020; Nishida et al. 2004; Watts 2000) and bonobos (e.g., Girard‐Buttoz et al. 2020; Sakamaki 2013; Surbeck and Hohmann 2015), relatively little is known about mother–infant interactions despite their crucial role in early development. Our results showed that infant‐mother grooming frequency increased with infant age, supporting limited research on infant‐mother grooming in bonobos and chimpanzees (e.g., Nishida 1988; van Lawick‐Goodall 1967). Similar patterns have been reported in other primate species (i.e., captive baboons (*Papio anubis*) and rhesus macaques), where grooming replaced physical carrying (Nash 1978), emphasizing the importance of tactile engagement in promoting and nurturing social bonds and trust (Watts 2000). Similarly, in humans, tactile interactions—including cuddling, playful touch, and hair‐brushing—strengthen attachment and emotion regulation, serving as a foundation for broader social skills beyond immediate caregivers (Phillips and Shonkoff 2000). Although prior studies have suggested species‐specific grooming dynamics, such as longer grooming sessions in subadult and adult bonobos (Girard‐Buttoz et al. 2020; Sakamaki 2013), our findings do not support these differences at the mother–infant level.

### 4.4 Spatial Independence

We found a species difference in spatial independence, with chimpanzees remaining closer to their mothers across early development compared to bonobos, despite both species showing similar age‐related developmental trajectories. This contrasts with findings suggesting that bonobos maintain closer maternal proximity and develop more slowly (De Lathouwers and Van Elsacker 2006; Fröhlich et al. 2016; Koops et al. 2015), but these rarely involved direct, systematic comparisons. Our findings align with results of Lee and colleagues (2020), who reported that bonobo infants spent more time at greater distances from their mothers compared to chimpanzees, albeit only beyond the age of 3. Our data show a similar trend: at 3.5 years, bonobo infants were more than 5 m away from their mothers in over 50% of scans, increasing to 80% by age 5. Chimpanzees reached comparable levels only around 4.5 years (50%), and 60% by age 5. Factors beyond developmental pace, such as exposure to danger and maternal styles, may also shape mother–infant proximity patterns. While aggression levels and intensity towards immatures appear similar in both species (e.g., Furuichi 1997; Hohmann et al. 2019; Reddy et al. 2024), only chimpanzees show lethal aggression and infanticide (Arcadi and Wrangham 1999; Watts and Mitani 2000; Wilson et al. 2014). This heightened risk in chimpanzees may explain why infants and mothers remain closer together. Additionally, chimpanzee mothers at Ngogo provided more support during aggression than bonobo mothers at Kokolopori (Reddy et al. 2024), potentially explaining why bonobo infants venture farther while chimpanzee infants stay closer. Similar patterns occur in other apes and populations: Bornean orangutan (*Pongo pygmaeus*) mothers actively reduce distance to offspring in the presence of males (Scott et al. 2023), and chimpanzee infants at Kanyawara, Uganda, moved farther from their mothers when fewer males were present (Otali and Gilchrist 2006). While predator pressure can also affect proximity (e.g., Förster and Cords 2002), this seems unlikely since most leopard populations have been eliminated in our study populations (Watts 2008; Surbeck, personal communication). Nevertheless, species differences in proximity patterns may reflect evolutionary adaptations to historical predation or other environmental pressures that continue to shape behaviour even when the original selective forces are no longer present.

Although proximity patterns differed between bonobos and chimpanzees, our findings underscore a broader developmental trend: in both species, infants remain in close maternal proximity for extended periods, highlighting the prolonged dependence of both great ape species on maternal care. Our results align with investigations of general social behaviour in human and nonhuman primates, with infants gradually reducing proximity to their mothers and caregivers during development (e.g., Nash 1978). While acknowledging substantial variation across human societies, some studies of hunter‐gatherer groups (e.g., the Ache, Aka, and Hadza) have documented that young children stay physically close (usually within 1 m; Konner 2017) to caregivers until about 4 to 5 years of age, transitioning gradually to more independent exploration while remaining under supervision (Broesch et al. 2021; Konner 2017).

### 4.5 Outlook

Our findings offer new insights into great ape development by using consistent parameters across two great ape species living in two different populations and considering both sexes. Several methodological improvements could further enhance our understanding of developmental trajectories and maternal strategies in *Pan*. Expanding research across broader age ranges is essential, as behaviours observed up to age 5 may persist beyond this period, underscoring the need for longitudinal studies covering extended developmental windows. Additionally, our findings emphasize the fluid nature of behavioural transitions, underscoring the importance of examining development continuously rather than categorizing behaviours by rigid age groups, which may overlook nuanced developmental aspects. Beyond methodological considerations, expanding the scope to include interactions of infants with siblings, peers and other adult individuals, as well as variation across communities, will provide a more holistic understanding of great ape social development over time. Large‐scale collaboration among researchers across multiple field sites and institutions has been instrumental in advancing our understanding of primate development, with initiatives such as 1000PAN (https://www.comparative‐biocognition.de/1000pan/about‐1000pan) and ManyPrimates (Primates et al. 2019) offering promising avenues for integrating datasets and improving comparability across populations and species. Our findings also have important implications for ongoing theoretical debates about bonobo social evolution, suggesting that the complex developmental pathways we observed, shaped by multiple ecological and social factors rather than simple species‐level differences, may require reconsidering or refining the self‐domestication hypothesis. Finally, the extensive variability in maternal care patterns observed within primate species suggests that comparative studies between humans and non‐human primates should be interpreted carefully, particularly when human data derive from narrow cultural contexts (Amici et al. 2025). Moving forward, addressing discrepancies across studies through enhanced collaboration networks and data integration will be essential for building a more unified framework for primate ontogeny.

## 5 Conclusion

This study adds to the recent debate on species differences in the two Pan species by providing a systematic, quantitative assessment of developmental trajectories of bonobo and chimpanzee infants across two populations in the wild. Through longitudinal observations and cross‐species comparisons, we specifically focused on behavioural and spatial markers of independence, as these capture the transition from maternal reliance to early autonomy. The results concerning the first research question showed that developmental differences did not simply reflect species differences but appeared to be shaped by complex interactions between ecological pressures, social dynamics, and maternal strategies. The results concerning our second research question of spatial independence from mothers showed that there was a clear difference between bonobo and chimpanzee infants over time. Specifically, our results revealed that core developmental processes—ventral riding, nursing, and grooming—were characterised by considerable consistency between species, suggesting shared evolutionary constraints on mother–infant interactions. However, we found clear species differences concerning travel independence and spatial proximity, which may reflect distinct adaptive strategies shaped by differences in social risk, habitat structure, and maternal styles. Differences in rates of dorsal riding, however, appeared to reflect population‐level variation rather than species‐wide patterns, as chimpanzee infants in our study showed higher dorsal riding rates at older ages compared to bonobos—contrasting with one other population where mothers' strategies for managing multiple offspring differed. Our results thus indicate a mosaic pattern, with some developmental features being conserved (e.g., ventral riding, nursing, grooming) while others exhibit higher behavioural flexibility and plasticity (e.g., travel independence, spatial proximity). This mosaic pattern parallels genomic findings showing that humans share some derived traits exclusively with bonobos and others exclusively with chimpanzees (Prüfer et al. 2012), reinforcing that neither *Pan* species alone represents the ancestral condition. Rather than identifying which species is the better model, our findings underscore the necessity of comparing both *Pan* species to understand the range of developmental possibilities that may have characterized our last common ancestor and inform accounts of the developmental variation that likely characterized early hominin populations (e.g., Gruber and Clay 2016; Kuzawa and Bragg 2012). Critically, our findings demonstrate that individual‐level factors—such as maternal experience and sibling presence—operate similarly across species, indicating conserved mechanisms underlying developmental plasticity in *Pan*. These findings highlight the importance of studying multiple populations to better understand the ecological and social factors driving primate behavioural diversity. Rather than focusing on one species as a better comparative model, these findings reinforce the need to study both species to gain a more comprehensive understanding of human ontogeny and the shared features underlying primate development. By disentangling species‐specific traits from population‐level variability, we can deepen our understanding of the evolutionary origins of life history traits and their relevance to human development.

## Author Contributions

**Jolinde M. R. Vlaeyen**: data curation, formal analysis, investigation, methodology, writing – original draft, writing – review and editing. **Bas van Boekholt**: investigation, methodology, writing – review and editing. **Franziska Wegdell**: investigation, writing – review and editing. **Raymond Katumba**: investigation, writing – review and editing. **Andreas Berghänel**: formal analysis, writing – review and editing. **Martin Surbeck**: data curation, funding acquisition, methodology, supervision, project administration, writing and shaping of manuscript, writing – review and editing. **Simone Pika**: conceptualization, funding acquisition, methodology, supervision, project administration, writing and shaping of manuscript, writing – review and editing.

## Ethics Statement

The present study was purely observational and non‐invasive. It strictly adhered to all applicable national, and institutional guidelines for the care and use of animals, the ‘Animals (Scientific Procedures) Act 1986,’ and the American Society of Primatologists’ ‘Ethical Treatment of Non‐Human Primates.’ Classified as a non‐animal experiment under the German Animal Welfare Act (May 25, 1998, Section V, Article 7), no formal approval was required. Observers followed strict hygiene protocols, including quarantine, face masks (Kühl 2008), and a minimum observation distance of 7m to minimize disease transmission and behavioural disruption (e.g., Köndgen et al. 2008). Permits were obtained from Uganda Wildlife Authority (EDO‐35‐01), Uganda National Council for Science and Technology (NS488), Uganda, the Institut Congolais pour la Conservation de la Nature, and the Ministry of Research, DRC. Access to the Kokolopori Bonobo Reserve was granted by the communities of Bekungu, Bolamba, Yasalakose, Yetee and Yomboli.

## Conflicts of Interest

The authors declare no conflicts of interest.

## Supporting information



Supporting File 1: desc70102‐sup‐0001‐SuppMat.docx

## Data Availability

The dataset used in this study is openly accessible on Figshare at https://figshare.com/s/91aa961a1d0295fe8806.
