# Supplementary material for: Great Ape Childhoods: Social and Spatial Pathways to Independence in Bonobo and Chimpanzee Infants
Source: Dev Sci. 2025 Dec 15;29(1):e70102. doi: 10.1111/desc.70102 (PMC12703578; doi:10.1111/desc.70102)
Supplement: Supplementary file 1 — Supporting File 1: desc70102‐sup‐0001‐SuppMat.docx [file DESC-29-e70102-s001.docx]

**Supplementary materials for:
Great Ape Childhoods: Social and Spatial Pathways to Independence in Bonobo and Chimpanzee Infants**

**Authors:** Jolinde M.R. Vlaeyen^1*^, Bas van Boekholt^1^, Franziska Wegdell^2,3^, Raymond Katumba^4^, Andreas Berghänel^5,8^, Martin Surbeck^6,7^ and Simone Pika^1*^

***Corresponding authors:** Jolinde Vlaeyen: jolinde.vlaeyen@[uni-osnabrueck.de](mailto:simone.pika@uni-osnabrueck.de);  Simone Pika: [simone.pika@uni-osnabrueck.de](mailto:simone.pika@uni-osnabrueck.de)

**Affiliations:**

^1^ Institute of Cognitive Science, Comparative BioCognition, Osnabrück University, Germany
^2^ Department of Evolutionary Anthropology, University of Zurich, Zurich, Switzerland

^3^ Institute for the Interdisciplinary Study of Language Evolution, University of Zurich, Zurich, Switzerland

^4^ Sebitoli Chimpanzee Project, Great Ape Conservation Project, Fort Portal, Uganda

^5^ Department of Interdisciplinary Life Sciences, University of Veterinary Medicine, Vienna, Austria

^6^ Department of Human Evolutionary Biology, Harvard University, Cambridge, MA, USA.

^7^ Department of Human Behaviour, Ecology and Culture, Max Planck Institute for Evolutionary Anthropology, Leipzig, Germany
^8^ Zoology and Animal Ecology Research Group, Department of Biology, University of Hildesheim, Germany

**Supplementary materials**

**S1. Study communities**

***Bonobo communities***

The FKK community, with a total of nine members, included two infants (one male, one female), the EKK community, with 18–19 members, included five infants (three males, two females), and the KKL community, with 34–37 members, included 14 infants (five males, nine females). The KKL community is often split into two sub-communities (KKL South and KKL North), but were pooled together for the purpose of this study.

***Chimpanzee communities***

During the first study period (February–September 2021), the West community, with a total of 83–85 members, included 12 infants (seven males, five females), and the Central community, with 113–122 members, included seven infants (three males, four females). During the second study period (August 2022–February 2023), the West community, with 90–97 members, included 13 infants (seven males, six females), and the Central community, with 95–104 members, included five infants (two males, three females).

Table 1. *Overview of focal individuals and their respective date of birth, sex, mother, and species****.***

| **Focal Individuals** | **Date of birth** | **Sex** | **Community** | **Mother** | **Species** |
| --- | --- | --- | --- | --- | --- |
| Bly | 2021-11-21 | M | West | Binoche | Chimpanzee |
| Carol | 2019-01-29 | F | West | Renee | Chimpanzee |
| Colton | 2020-09-21 | F | Central | EmmyLou | Chimpanzee |
| Dorothy | 2018-01-15 | F | West | Rusalka | Chimpanzee |
| E.O. | 2017-12-05 | M | West | Carson | Chimpanzee |
| Gatsby | 2018-10-04 | M | Central | Fitzgerald | Chimpanzee |
| Goblin* | 2019-10-14 | M | Central | Hester | Chimpanzee |
| Haldane | 2020-01-02 | M | West | Beecher | Chimpanzee |
| Hubble | 2018-03-18 | F | Central | Violetta | Chimpanzee |
| Isobel* | 2020-10-11 | F | Central | Christine | Chimpanzee |
| Kano | 2018-03-08 | M | Central | Callas | Chimpanzee |
| Lecter | 2019-11-04 | M | West | Penelope | Chimpanzee |
| Leo | 2020-05-15 | M | West | Ntwiga | Chimpanzee |
| Lindsay | 2018-01-17 | F | Central | Beryl | Chimpanzee |
| Lootus | 2020-07-26 | F | West | Leigh | Chimpanzee |
| Louis | 2018-01-25 | M | West | Sabin | Chimpanzee |
| Malaika | 2018-12-28 | F | West | Miliah | Chimpanzee |
| MF DOOM | 2019-09-20 | M | West | Fleming | Chimpanzee |
| Oswald | 2022-04-24 | M | West | Carson | Chimpanzee |
| Sally | 2019-10-07 | F | West | Bacall | Chimpanzee |
| Sebas | 2022-08-10 | M | West | Sabin | Chimpanzee |
| Ursula | 2022-05-02 | F | West | Senta | Chimpanzee |
| Abba | 2018-04-06 | F | KKL | Adele | Bonobo |
| Amethyst | 2019-04-21 | M | EKK | Azur | Bonobo |
| Arda | 2021-07-08 | F | FKK | Amazonia | Bonobo |
| Curtis | 2018-08-04 | F | KKL | Chapman | Bonobo |
| Emeraude | 2020-05-01 | M | EKK | Eben | Bonobo |
| Enigma | 2019-06-04 | F | KKL | Elliot | Bonobo |
| Falco | 2018-05-25 | M | KKL | Fitz | Bonobo |
| Gwen | 2022-03-21 | F | KKL | Gloria | Bonobo |
| Kravitz | 2021-12-23 | M | KKL | Kidjio | Bonobo |
| Missouri | 2020-02-01 | M | FKK | Mississippi | Bonobo |
| Muse | 2018-12-31 | F | KKL | Madonna | Bonobo |
| Orange | 2020-05-01 | F | EKK | Olive | Bonobo |
| Ozzy | 2019-05-05 | F | KKL | Oliday | Bonobo |
| Piaf | 2018-08-03 | F | KKL | PJ | Bonobo |
| Prince | 2022-02-23 | M | KKL | PJ | Bonobo |
| Rubin | 2019-06-20 | M | EKK | Rose | Bonobo |
| Schubert | 2017-02-05 | M | KKL | Simone | Bonobo |
| Shakira | 2022-09-13 | F | KKL | Simone | Bonobo |
| Tango | 2022-05-19 | F | KKL | Tyler | Bonobo |
| Tupac | 2017-03-12 | M | KKL | Tyler | Bonobo |
| Vert | 2021-06-24 | F | EKK | Violette | Bonobo |

***** Died in between field seasons

**S2. Additional Methodological Details and Model Summaries**

***Inter-rater reliability:***

Since the cooperation project began after data collection was completed, an inter-rater reliability test could not be conducted for the bonobo dataset. However, JV and FW discussed all definitions of their scan data in detail to ensure consistency and comparability of collected data and subsequent analyses. For the chimpanzee dataset, inter-rater reliability was assessed between BvB and RK for both scan and focal data. For proximity categories in the scan data (n = 51), Cohen’s Kappa values were calculated separately for each distance bin: κ = 0.697 for <1 m, κ = 0.406 for 1–5 m, and κ = 0.827 for >5 m, yielding an overall weighted Kappa of 0.697. According to the interpretation scale proposed by Landis and Koch (1977), this reflects **substantial agreement** overall, with **almost perfect agreement** for the >5 m category and **moderate agreement** for 1–5 m. The lower agreement in the intermediate category likely reflects its inherent ambiguity and low frequency. For focal data, which included continuously recorded behaviors, agreement was assessed by comparing independently recorded behavior counts during four sessions occurring on three days of simultaneous observation. Observers showed high agreement for key behaviors, including identical counts for nursing (15 instances each), and minimal differences for grooming (5 vs. 6) and ventral riding (5 vs. 4). Discrepancies were greater for dorsal riding (57 vs. 42) and independent travel (0 vs. 2), likely due to their short duration or ambiguity in boundary definitions. Overall, these results indicate substantial reliability for most key behaviors in the chimpanzee dataset (Landis & Koch, 1977).

***Model Assumptions and stability:***

Overdispersion was not a problem for most models (Model1a: 0.52; Model1b: 0.92; Model1c: 0.41; Model2: 0.71; Model3: 0.74). For models with slight under dispersion, the residuals did not indicate strong deviations from model assumptions, and no alternative model structure substantially improved model fit. After fitting the model, we assessed model assumptions by visually inspecting QQ plots of residuals (Field, 2005) and residuals plotted against fitted values (Quinn & Keough, 2002). These diagnostics did not indicate any violations of model assumptions. Finally, we assessed model stability by systematically excluding observations one at a time leave-one-out (LOO) analyses (Nieuwenhuis et al., 2012) and performed parametric bootstrapping (function “bootMer” of the package *lme4*; N=1000 bootstraps) to obtain confidence intervals of model estimates and fitted values. All models tested in this study demonstrated overall stability, with bootstrapped estimates producing consistent distributions across parameters. The density plots for bootstrapped estimates showed normal or near-normal distributions for most fixed effects, with no significant deviations suggesting instability. However, the variable of having a younger sibling presented some variability in its bootstrapped estimates, as indicated by a relatively high standard deviation and a wider range of values in comparison to other predictors. This variability is likely due to the unbalanced nature of the dataset, where fewer individuals have younger siblings, leading to greater uncertainty in the model estimates. Given that our dataset includes individuals up to 5.5 years old, it is expected that relatively few individuals had younger siblings. Despite this, additional diagnostic checks, including boxplots and density plots, confirmed that the estimates remained within a biologically plausible range and were not extreme outliers. While its effect size showed some variability, its inclusion significantly contributed to model fit in almost all cases, as evidenced by the likelihood ratio tests. Given the biological importance of sibling presence in shaping infant behaviour, we therefore retained this predictor in our models.

Additionally, Model 4 (Proximity) included rain and party size as random effects, but these variables had 900 missing data points. To assess their impact, we tested a model excluding these variables and found that, while this increased the sample size, it resulted in a substantially worse AIC, indicating a poorer model fit. Since the overall results remained unchanged, we opted to retain rain and party size as random intercepts in the final model, leading to the exclusion of 900 data points. Given the model's complexity, we also retained only the most relevant random effects in the final model to improve efficiency.

***Bootstrap analysis:***

To account for unequal sample sizes between species, we ran bootstrap analyses for Models 1, 2, and 4. Model 3 was not rebalanced because the number of observations per species was comparable. In each of 500 iterations, we down-sampled the larger species (without replacement) to match the number of observations in the smaller species, refitted the full model, and extracted the species effect estimates. For Models 1 and 2, we ensured each individual contributed at least three observations per iteration where possible; for Model 4, at least one observation per individual was included.

***Age calculations:***

To determine the average ages at which bonobos and chimpanzees start or stop exhibiting a specific behaviour, we first identified the age at which there is a 50% probability of the behaviour occurring, based on our observational data. This 50% probability mark served as a threshold to categorize individuals into two groups: those younger and those older than this threshold age. For each species, we then calculated the mean age for the individuals in each group (those below and those above the 50% probability mark).

**S3. Results**

1. **QUESTION ONE - General behavioural Patterns**
   1. ***TRAVEL MODES***

**VENTRAL RIDING**

***Descriptive results.*** Both species ceased ventral riding around 3 years (±1 year for both species). The last observed instance of ventral riding was at 4.3 years for bonobos (N=1) and 5 years for chimpanzees (N=1).

***Statistical Model Summary:***

Table 2. *Results of the GLMM examining factors influencing ventral riding in bonobo and chimpanzee infants. Age was z-scored and all fixed effects were centred before analysis. Significant effects (p < 0.05) are in bold. Total observations = 563 (Bonobo: 375; Chimpanzee: 188).*

|  | Estimate | Std. Error | z value | Pr(>\|z\|) | 95% CI |
| --- | --- | --- | --- | --- | --- |
| **Intercept** | **-1.0587** | **0.3366** | **-3.146** | **0.00166** | **[-1.718, -0.399]** |
| **Age** | **-2.5697** | **0.4855** | **-5.293** | **1.21E-07** | **[-3.521, -1.618]** |
| Species (Chimpanzee) | -0.4963 | 0.3803 | -1.305 | 0.19188 | [-1.242, 0.249] |
| Sex (Male) | -0.7324 | 0.3808 | -1.923 | 0.05448 | [-1.479, 0.014] |
| Younger Sibling Presence (Yes) | -0.7433 | 0.6032 | -1.232 | 0.21785 | [-1.926, 0.439] |
| **Mom Parity (Primiparous)** | **0.9821** | **0.4253** | **2.309** | **0.02092** | **[0.149, 1.816]** |

**Random Effects (SD):**

- Age slope (ID): 1.19
- Intercept (Mom_ID): 0.64
- Age slope (Group): 0.70
- Other variance components ≈ 0

Despite variation in how quickly infants reduced ventral riding (SD = 1.19), all individuals showed negative slopes, confirming a robust age-related decline. Bootstrap results confirmed that this pattern was robust to species sample-size imbalance: Species effect remained non-significant across iterations (mean = –0.32, 95% CI [–1.19, 0.58]).

**DORSAL RIDING**

***Descriptive results.*** Both species began dorsal riding around 1 year (bonobos: 1 ± 8 months; chimpanzees: 7 ± 3 months). The earliest instance was observed at 5 months for bonobos (N=1) and 4 months for chimpanzees (N=1). Bonobos continued dorsal riding until an average of 3.8 years (±5 months), while chimpanzees continued until an average of 4.9 years (±2 months). The last observed instance of dorsal riding was at 4.8 years for bonobos (N=1) and 5 years for chimpanzees (N=1).

***Statistical Model Summary:***

Table 3. *Results of the GLMM examining factors influencing dorsal riding in bonobo and chimpanzee infants. Age was z-scored and all fixed effects were centred before analysis. Significant effects (p < 0.05) are in bold. Total observations = 563 (Bonobo: 375; Chimpanzee: 188).*

|  | Estimate | Std. Error | z value | Pr(>\|z\|) | 95% CI |
| --- | --- | --- | --- | --- | --- |
| Intercept | 0.2523 | 0.3072 | 0.821 | 0.411455 | [-0.350, 0.855] |
| **Age** | **-1.1582** | **0.4946** | **-2.342** | **0.019196** | **[-0.350, 0.855]** |
| **Age^2** | **-2.1828** | **0.3411** | **-6.399** | **1.57E-10** | **[-0.350, 0.855]** |
| **Species (Chimpanzee)** | **1.7913** | **0.3355** | **5.34** | **9.29E-08** | **[1.134, 2.449]** |
| Younger Sibling Presence (Yes) | 0.4294 | 0.7023 | 0.611 | 0.540897 | [1.134, 2.449] |
| Sex (Male) | 0.2154 | 0.3238 | 0.665 | 0.50592 | [-0.419, 0.850] |
| **Mom Parity (Primiparous)** | **-1.0658** | **0.3832** | **-2.781** | **0.005415** | **[-1.817, -0.315]** |
| **Age:Species (Chimpanzee)** | **2.4301** | **0.6895** | **3.524** | **0.000425** | **[1.079, 3.782]** |
| **Age:Younger Sibling Presence (Yes)** | **-4.1595** | **1.7398** | **-2.391** | **0.016809** | **[-7.569, -0.750]** |

**Random Effects (SD):**

- Age slope (ID): 1.61
- Intercept (Mom_ID): 0.41
- Age slope (Group): 0
- Other variance components ≈ 0

Infants differed in the timing and shape of dorsal riding trajectories (SD = 1.61), but most followed the same curved developmental pattern. Bootstrap results confirmed that this pattern was robust to species sample-size imbalance: Strong positive species effect (mean = 1.75, 95% CI [1.04, 2.62]) remained significant in > 95% of iterations.

**INDEPENDENT TRAVEL & TRAVEL ON MOTHER**

***Descriptive results.*** Bonobos ceased riding on their mothers at an average of 4.4 years (±2 months) and chimpanzees a little later, at 4.8 years (±1 month). Independent travel began at approximately 4 years (±7.5 months) for bonobos and 4.7 years (±2 months) for chimpanzees. The last instances of riding on mother were observed around 5 years (4.6 for bonobos, 5 for chimpanzees, N=1) and the earliest instances of independent travel occurred at 11 months (bonobos, N=1) and 2.7 years (chimpanzees, N=1).

***Statistical Model Summary:***

Table 4. *Results of the GLMM examining factors influencing independent travel in bonobo and chimpanzee infants. Age was z-scored and all fixed effects were centred before analysis. Significant effects (p < 0.05) are in bold. Total observations = 563 (Bonobo: 375; Chimpanzee: 188).*

|  | Estimate | Std. Error | z value | Pr(>\|z\|) | 95% CI |
| --- | --- | --- | --- | --- | --- |
| **Intercept** | **-1.7069** | **0.6579** | **-2.594** | **0.00947** | **[-2.996, -0.417]** |
| **Age** | **5.1722** | **0.7809** | **6.623** | **3.52E-11** | **[3.642, 6.703]** |
| **Species (Chimpanzee)** | **-5.088** | **0.7542** | **-6.746** | **1.52E-11** | **[-6.566, -3.610]** |
| Sex (Male) | 1.0657 | 0.9186 | 1.16 | 0.246 | [-0.735, 2.866] |
| **Younger Sibling Presence (Yes)** | **2.5319** | **0.7822** | **3.237** | **0.00121** | **[0.999, 4.065]** |
| Mom Parity (Primiparous) | 0.383 | 0.5866 | 0.653 | 0.51383 | [-0.767, 1.533] |

**Random Effects (SD):**

- Age slope (ID): 2.55
- Intercept (Mom_ID): 0.35
- Age slope (Group): 0
- Other variance components ≈ 0

This model showed the widest individual variation (SD = 2.55), with steep increases in some infants and gradual transitions in others. Bootstrap results confirmed that this pattern was robust to species sample-size imbalance: Strong negative species effect (mean = –4.59, 95% CI [–5.57, –3.50]) remained significant across all iterations.

- 1. ***FEEDING MODES***

**NIPPLE CONTACT & INDEPENDENT FEEDING**

***Descriptive results.*** Both species had nipple contact until around 3.7–3.9 years (±4.5–7.5 months) and began independent feeding at approximately 2 years (bonobos: ±11 months; chimpanzees: ±7 months). The last observed nipple contact occurred at 4.5 years in bonobos (N=1) and 5 years in chimpanzees (N=1), while independent feeding began as early as 7 months in bonobos (N=1) and 6 months in chimpanzees (N=1).

***Statistical Model Summary:***

Table 5. *Results of the GLMM examining factors influencing nipple contact in bonobo and chimpanzee infants. Age was z-scored and all fixed effects were centred before analysis. Significant effects (p < 0.05) are in bold. Total observations = 516 (Bonobo: 164; Chimpanzee: 352).*

|  | Estimate | Std. Error | z value | Pr(>\|z\|) | 95% CI |
| --- | --- | --- | --- | --- | --- |
| Intercept | 0.4111 | 0.34694 | 1.185 | 0.236 | [-0.269, 1.091] |
| Species (Chimpanzee) | -0.55148 | 0.35464 | -1.555 | 0.1199 | [-1.247, 0.144] |
| Sex (Male) | -0.05473 | 0.3038 | -0.18 | 0.857 | [-0.650, 0.541] |
| **Age** | **-1.15972** | **0.23962** | **-4.84** | **1.30E-06** | **[-1.629, -0.690]** |
| **Younger Sibling Presence (Yes)** | **1.27914** | **0.61262** | **2.088** | **0.0368** | **[0.078, 2.480]** |
| Mom Parity (Primiparous) | 0.01319 | 0.35194 | 0.037 | 0.9701 | [-0.677, 0.703] |
| **Age:Younger Sibling Presence (Yes)** | **-4.15933** | **0.95799** | **-4.342** | **1.41E-05** | **[-6.037, -2.282]** |

**Random Effects (SD):**

- Age slope (ID): 0.94
- Intercept (Mom_ID): 0.60
- Age slope (Group): 0.15
- Other variance components ≈ 0

There was moderate variation in nipple contact trajectories (SD = 0.94), with some infants showing prolonged nipple contact and others declining more quickly. Bootstrap results confirmed that this pattern was robust to species sample-size imbalance: Species effect remained non-significant (mean = –0.31, 95% CI [–1.01, 0.56]).

- 1. **GROOMING**

***GROOMING***

***Descriptive results:*** On average, bonobos and chimpanzees start grooming their mothers around 1.9 (±1 year) and 2.7 (±10 months) years, respectively. The first time an infant was observed to groom their mother was at the age of 3.5 months in bonobos (N=1), and 1.2 years in chimpanzees (N=1).

***Statistical Model Summary:***

Table 6. *Results of the GLMM examining factors influencing grooming mothers in bonobo and chimpanzee infants. Age was z-scored and all fixed effects were centred before analysis. Significant effects (p < 0.05) are in bold. Total observations = 463 (Bonobo: 260; Chimpanzee: 203).*

|  | Estimate | Std. Error | z value | Pr(>\|z\|) | 95% CI |
| --- | --- | --- | --- | --- | --- |
| **Intercept** | **-1.56889** | **0.34218** | **-4.585** | **4.54E-06** | **[-2.240, -0.898]** |
| Species (Chimpanzee) | -0.28681 | 0.3973 | -0.722 | 0.470 | [-1.066, 0.492] |
| Sex (Male) | -0.08646 | 0.35491 | -0.244 | 0.808 | [-0.782, 0.609] |
| **Age** | **1.10638** | **0.22312** | **4.959** | **7.09E-07** | **[0.669, 1.544]** |
| Younger Sibling Presence (Yes) | 0.67986 | 0.50628 | 1.343 | 0.179 | [-0.312, 1.672] |
| Mom Parity (Primiparous) | 0.24204 | 0.73389 | 0.33 | 0.742 | [-1.196, 1.680] |

**Random Effects (SD):**

- Age slope (ID): 0
- Intercept (Mom_ID): 0.61
- Age slope (Group): 0.79
- Other variance components ≈ 0

No individual variation in developmental slope was retained (SD ≈ 0), indicating a highly uniform increase in grooming with age.

Table 7. *Table summarizing the developmental timelines based on our descriptive statistics for different behaviours in bonobos and chimpanzees observed in this study. Mean start or end, as well as first and last times the behaviours were observed are included.* *Riding on mother includes both ventral and dorsal riding, hence the different mean end time compared to dorsal riding. (Y=years, M=months)*

|  |  | **BONOBO** | | **CHIMPANZEE** | |
| --- | --- | --- | --- | --- | --- |
|  | **Behaviour** | **Start ± SD** | **End ± SD** | **Start ± SD** | **End ± SD** |
| **TRAVEL** | **Ride on mother°** | 0y | 3.4y (±11m) *Last:* 4.6y | 0y | 4.8y (±1.5m) *Last:* 5y |
|  |  | *Earlier than LuiKotale^(1)^, similar to Wamba^(2)^* | | *In line with Gombe ^(1,3)^* | |
|  | **Ventral** | 0y | 3.1y (±9m) *Last:* 4.25y | 0y | 3.25y (±1y)  *Last:* 5y |
|  |  | *Later than Wamba^(2)^* | | *Later than Gombe^(3)^* | |
|  | **Dorsal*** | 1y (±8m)  1st: 5m | 3.8y (±5m) *Last:* 4.8y | 7m (±3m) *1st:* 4.2m | 4.9y (±2m)  *Last:* 5y |
|  |  | *Earlier onset than Wamba^(2)^* | | *Comparable onset to Gombe and Tai^(2,3,4)^* | |
|  | **Independent Travel** | 4y (±7.5m) *1st:* 11m | ∞ | 4.7y (±2m) *1st:* 2.7y | ∞ |
|  |  | *Not reported elsewhere* | | *Not reported elsewhere* | |
| **FEEDING** | **Nipple contact** | 0y | 3.7y (±4.5m) *Last:* 4.5y | 0y | 3.9y (±7.5m) *Last:* 5y |
|  |  | *Earlier than most sites ^(1,2,5)^* | | *In the normal range ^(6,7,8)^* | |
|  | **Independent feeding°** | 2y (±11m) *1st:* 7m | ∞ | 1.25y (±7m) *1st:* 6m | ∞ |
|  |  | *Similar to other sites ^(2)^* | | *Early onset aligns with Tai ^(4)^* | |
|  | **Grooming** | 1.9y (±1y)  *1st:* 3.5m | ∞ | 2.7y (±10m) *1st:* 1.2y | ∞ |
|  |  | *Earlier than peer grooming ^(1)^* | | *Comparable with peer grooming ^(4)^* | |

**Note:** °Not analysed statistically, as inverse binomial data of *Independent Travel* and *Nursing*, respectively; *Only statistical difference.
1. Lee et al. (2020); 2. Kuroda (1989); 3. van Lawick-Goodall (1967); 4. Bründl et al. (2021); 5. De Lathouwers and Van Elsacker (2006); 6. Oelze et al. (2024); 7. Bădescu et al. (2017); 8. Lonsdorf et al. (2020).

1. **QUESTION TWO – Spatial independence patterns**

**SPATIAL INDEPENDENCE**

**Statistical Model Summary:**

Table 8. *Results of the CLMM examining factors influencing distance to mothers in bonobo and chimpanzee infants. Age was z-scored and all fixed effects were centred before analysis. Significant effects (p < 0.05) are in bold. Total observations = 5,006 (Bonobo: 643; Chimpanzee: 4,361). Threshold coefficients are under the dotted line.*

|  | Estimate | Std. Error | z value | Pr(>\|z\|) | 95% CI |
| --- | --- | --- | --- | --- | --- |
| **Age** | **1.4645** | **0.1391** | **10.527** | **< 2e-16** | [1.192, 1.737] |
| **Species (Chimpanzee)** | **-1.133** | **0.2345** | **-4.831** | **1.36E-06** | [-1.593, -0.673] |
| Sex (Male) | -0.2847 | 0.2177 | -1.308 | 0.191 | [-0.712, 0.142] |
| Age | -0.2103 | 0.2415 | -0.871 | 0.384 | [-0.684, 0.263] |
| Mom Parity (Primiparous) | 0.0676 | 0.2523 | 0.268 | 0.789 | [-0.427, 0.562] |
| *<1m\|1-5m* | 5.0213 | 0.3386 | 14.83 |  | [4.358, 5.685] |
| *1-5m\|>5m* | 6.4206 | 0.341 | 18.83 |  | [5.752, 7.089] |

**Random Effects (SD):**

- Age slope (ID): 0.58
- Intercept (Mom_ID): 0.43
- Other variance components ≈ 0

Moderate variation in slope (SD = 0.58) suggests infants differed in how quickly they increased distance from their mothers, but the overall developmental trend remained consistent. Bootstrap results confirmed that this pattern was robust to species sample-size imbalance: Species effect (mean = –1.33, 95% CI [–1.58, –1.11]) remained highly significant.


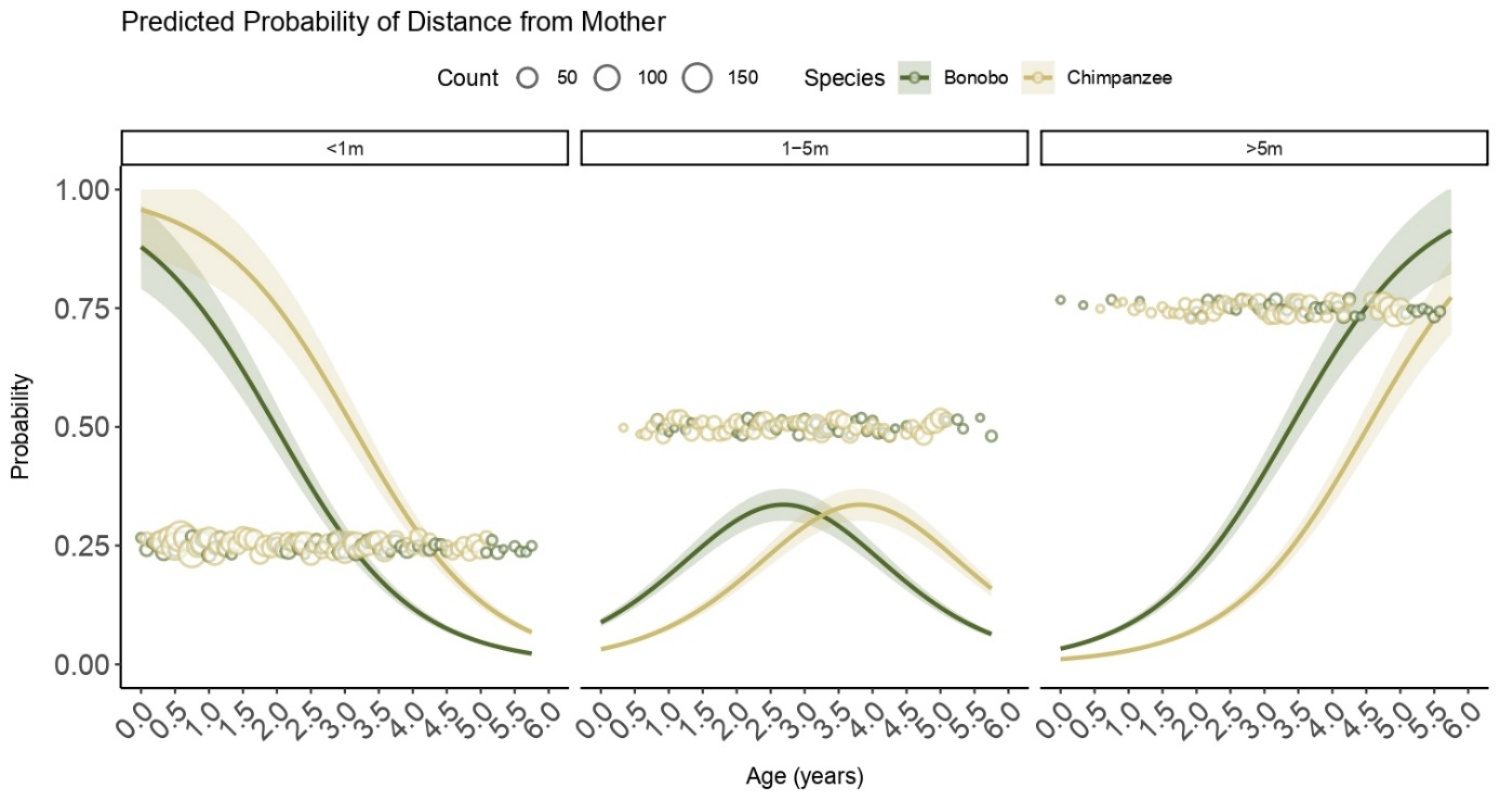


*Figure 1.* Predicted probabilities of an infant’s distance from its mother at different ages (in years) in bonobos (dark green) and chimpanzees (yellow), including the 95% asymptotic confidence intervals. Bubbles depict the raw data with bubble area being proportional to the number of observations for each infant/age combination, including the offset. The percentages lines are not representative of the data.

**S4. Additional results**

For bonobos, each time data was collected on whether infants travelled ventrally, dorsally, or independently, additional data were also collected on the substrate they were travelling on, namely climbing up or down, travelling in the trees, and travelling on the ground. This was not done for the chimpanzee data, and thus we have no comparable data.

| ***Ways of travelling*** | **Climb Down Tree** | **Climb Up Tree** | **Travel in Trees** | **Travel on Ground** |
| --- | --- | --- | --- | --- |
| **Travel on mother** | 196 | 111 | 153 | 705 |
| Ventral Riding | 159 | 79 | 135 | 220 |
| Dorsal Riding | 37 | 32 | 18 | 485 |
| **Independent Travel** | 107 | 85 | 253 | 133 |

| ***Ways of travelling*** | **% Travel Trees** | **% Travel Ground** |
| --- | --- | --- |
| *Including climb up & down* | | |
| **Travel on mother** | 39.5 | 60.5 |
| **Independent Travel** | 77.0 | 23.0 |
| *Excluding climb up & down* | | |
| **Travel on mother** | 17.8 | 82.2 |
| **Independent Travel** | 65.5 | 34.5 |


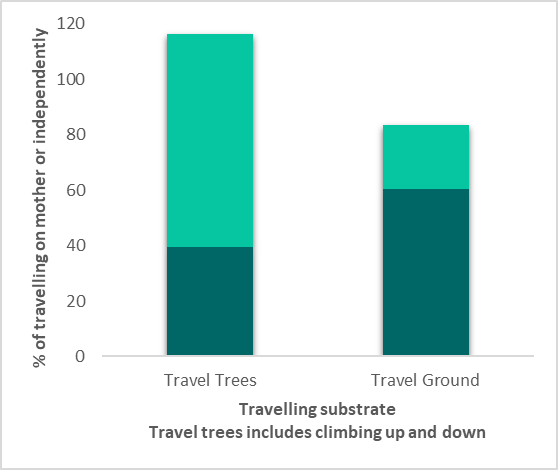


a


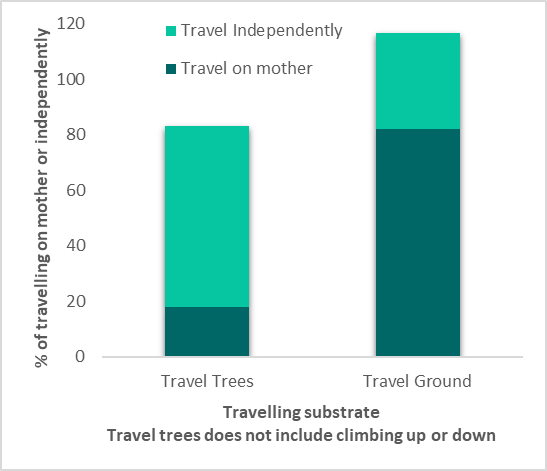


b.

*Figure 2.* Figures showing the percentage of times infant bonobos were seen travelling on their mothers compared to travelling independently on different substrates, either in the trees or on the ground. Figure 2a represents the data when including the categories “climbing up the tree” and “climbing down the tree”, whereas Figure 2b represents the data when excluded said categories.

**REFERENCES:**

Bădescu, I., Katzenberg, M. A., Watts, D. P., & Sellen, D. W. (2017). A novel fecal stable isotope approach to determine the timing of age-related feeding transitions in wild infant chimpanzees. *Am J Phys Anthropol*, *162*(2), 285-299. <https://doi.org/10.1002/ajpa.23116>

Bründl, A. C., Tkaczynski, P. J., Nohon Kohou, G., Boesch, C., Wittig, R. M., & Crockford, C. (2021). Systematic mapping of developmental milestones in wild chimpanzees. *Developmental science*, *24*(1), e12988.

De Lathouwers, M., & Van Elsacker, L. (2006). Comparing infant and juvenile behavior in bonobos (Pan paniscus) and chimpanzees (Pan troglodytes): a preliminary study. *Primates*, *47*, 287-293.

Kuroda, S. (1989). Developmental retardation and behavioral characteristics of pygmy chimpanzees. In *Understanding chimpanzees* (pp. 184-193). Harvard University Press.

Lee, S. M., Murray, C. M., Lonsdorf, E. V., Fruth, B., Stanton, M. A., Nichols, J., & Hohmann, G. (2020). Wild bonobo and chimpanzee females exhibit broadly similar patterns of behavioral maturation but some evidence for divergence. *American journal of physical anthropology*, *171*(1), 100-109.

Lonsdorf, E. V., Stanton, M. A., Pusey, A. E., & Murray, C. M. (2020). Sources of variation in weaned age among wild chimpanzees in Gombe National Park, Tanzania. *American journal of physical anthropology*, *171*(3), 419-429.

Nieuwenhuis, R., Grotenhuis, M. t., & Pelzer, B. (2012). Influence. ME: Tools for detecting influential data in mixed effects models.

Oelze, V. M., Ott, K., Lee, S. M., O'Neal, I., Hohmann, G., & Fruth, B. (2024). Preliminary isotopic assessment of weaning in bonobos shows evidence for extended nursing, sibling competition and invested first‐time mothers. *American journal of primatology*, *86*(11), e23678.

van Lawick-Goodall, J. (1967). Mother-offspring relationships in free-ranging chimpanzees. In *Primate ethology* (pp. 287-346). Routledge.
